# Discovery and Characterization of Novel Antagonists of the Proinflammatory Orphan Receptor GPR84

**DOI:** 10.1021/acsptsci.1c00151

**Published:** 2021-09-07

**Authors:** Laura Jenkins, Sara Marsango, Sarah Mancini, Zobaer Al Mahmud, Angus Morrison, Stuart P. McElroy, Kirstie A. Bennett, Matt Barnes, Andrew B. Tobin, Irina G. Tikhonova, Graeme Milligan

**Affiliations:** † The Centre for Translational Pharmacology, Institute of Molecular, Cellular and Systems Biology, College of Medical, Veterinary and Life Sciences, University of Glasgow, Glasgow G12 8QQ, United Kingdom; ‡ BioAscent Discovery Ltd., Bo’Ness Road, Newhouse, Lanarkshire ML1 5UH, United Kingdom; § Sosei Heptares, Steinmetz Building, Granta Park, Great Abington, Cambridge CB21 6DG, United Kingdom; ∥ School of Pharmacy, Medical Biology Centre, Queen’s University Belfast, Belfast BT9 7BL, United Kingdom

**Keywords:** G protein-coupled receptor, species orthologues, immune cells, GPR84, high throughput screening, molecular modeling

## Abstract

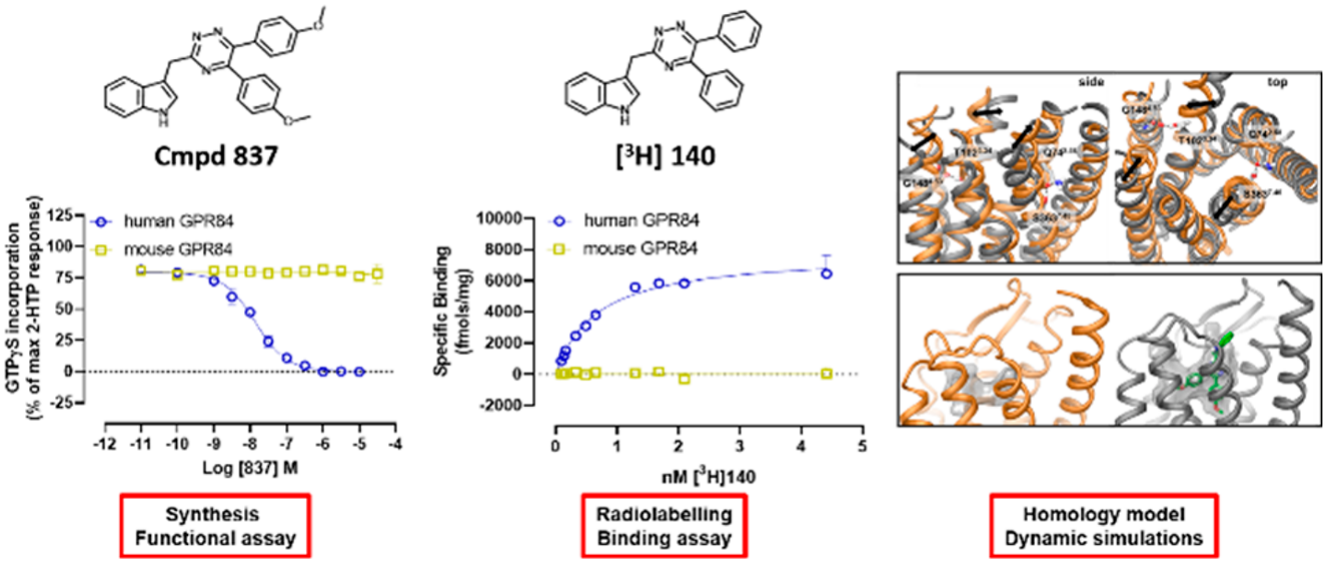

GPR84 is a poorly characterized, nominally orphan, proinflammatory G protein-coupled receptor that can be activated by medium chain length fatty acids. It is attracting considerable interest as a potential therapeutic target for antagonist ligands in both inflammatory bowel diseases and idiopathic pulmonary fibrosis. Successful screening of more than 300 000 compounds from a small molecule library followed by detailed analysis of some 50 drug-like hits identified 3-((5,6-bis(4-methoxyphenyl)-1,2,4-triazin-3-yl)methyl)-1*H*-indole as a high affinity and highly selective competitive antagonist of human GPR84. Tritiation of a di-iodinated form of the core structure produced [^3^H]3-((5,6-diphenyl-1,2,4-triazin-3-yl)methyl)-1*H*-indole, which allowed effective measurement of receptor levels in both transfected cell lines and lipopolysaccharide-treated THP-1 monocyte/macrophage cells. Although this compound series lacks significant affinity at mouse GPR84, homology modeling and molecular dynamics simulations provided a potential rationale for this difference, and alteration of two residues in mouse GPR84 to the equivalent amino acids in the human orthologue, predicted to open the antagonist binding pocket, validated this model. Sequence alignment of other species orthologues further predicted binding of the compounds as high affinity antagonists at macaque, pig, and dog GPR84 but not at the rat orthologue, and pharmacological experiments confirmed these predictions. These studies provide a new class of GPR84 antagonists that display species selectivity defined via receptor modeling and mutagenesis.

Although medium chain length fatty acids (MCFAs) have been known for a considerable period of time to be able to activate the G protein-coupled receptor (GPCR) GPR84,^1−3^ this receptor remains classified as an “orphan”, i.e. that the true activator(s) of the receptor remain undefined or uncertain.^4^ Expressed by a variety of immune cells, including monocytes, macrophages, and neutrophils in the periphery, and microglia in the brain^2,3,5^ as well as tissues such as adipocytes^6^ and skeletal muscle,^7^ GPR84 has recently attracted considerable interest as a therapeutic target. In significant part, this reflects that GPR84 transcript and protein level is upregulated in many proinflammatory conditions and directly in response to proinflammatory stimuli.^1,8−10^ This suggests that blocking the receptor might have value both in limiting the development of inflammatory conditions and during their resolution. Despite the identification and study of a significant number of predominantly lipid-like synthetic compounds that have moderate to high agonist potency at GPR84,^2,11^ the availability and variety of high affinity GPR84 antagonists is much less extensive. Indeed, the only widely available high affinity GPR84 antagonist is 9-cyclopropylethynyl-2-((*S*)-1-[1,4]dioxan-2-ylmethoxy)-6,7-dihydropyrimido[6,1-*a*]isoquinolin-4-one (GLPG1205).^12^ This compound reduced disease activity index score and neutrophil infiltration in a mouse dextran sodium sulfate-induced chronic inflammatory bowel disease model^12^ but failed to achieve efficacy end points in clinical trials in ulcerative colitis.^12^ By contrast, GLPG1205 has been reported to have positive effects in mouse models of lung fibrosis^13^ and is currently undergoing clinical trials in patients with idiopathic pulmonary fibrosis (ClinicalTrials.gov Identifier: NCT03725852), where it has shown an ability to lower lung function decline in adults. These encouraging findings indicate that the availability of a broader range of high affinity GPR84 antagonists would be of considerable use in better understanding the therapeutic potential to block this receptor in a range of conditions and might offer distinct starting points for novel medicines. Herein, we report the discovery and characterization of a novel series of high affinity and selective antagonists of GPR84. In early studies, we noted that these molecules were not active at mouse GPR84. However, from molecular modeling in conjunction with mutagenesis, we delineate the molecular basis for human versus mouse orthologue selectivity and subsequently confirm the predicted activity or otherwise of this compound series at other species orthologues of GPR84.

## Results

### Initial Screens

**Figure 1:**
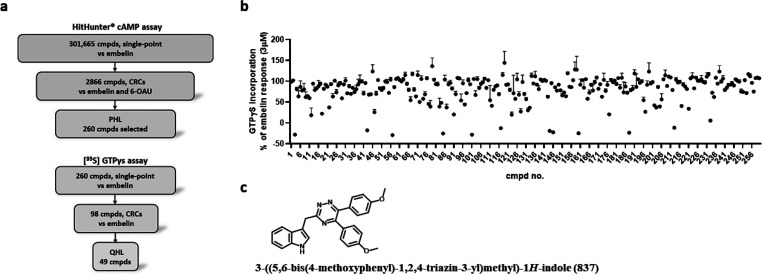
High throughput screening identifies a novel and potent GPR84 antagonist. Ability of each of 301 665 compounds to prevent embelin-mediated regulation of cAMP levels in CHO cells expressing human GPR84 was assessed (a). 2866 compounds were taken forward into concentration–response curves (CRCs) against both embelin and 6-OAU. Chemical- and potency-based triage selected 260 compounds that formed the preliminary hit list (PHL) (a). These compounds were further assessed in [^35^S]GTPγS binding assays performed on membranes of Flp-In TREx 293 cells induced to stably express a human GPR84-Gα_i2_ fusion protein (a, b). 98 compounds were taken forward into concentration–response studies, and 49 designated a QHL (a). 3-((5,6-Diphenyl-1,2,4-triazin-3-yl)methyl)-1*H*-indole (compound 837) was resynthesized for detailed study (c).

GPR84 is known to interact effectively and selectively with pertussis toxin-sensitive G_i_-family G proteins and hence reduce levels of cAMP in cells.^1^ In partnership with the European Lead Factory (https://www.europeanleadfactory.eu/) we successfully screened 301 665 drug-like small molecules^14^ against human GPR84 expressed stably in Chinese Hamster Ovary (CHO) cells (Figure 1a). A positive end point was suppression of the capacity of the GPR84 agonist 2,5-dihydroxy-3-undecyl-1,4-benzoquinone (embelin)^2,11,15,16^ to inhibit forskolin-mediated elevation of cAMP levels. Initial hits that passed threshold were then retested in concentration–response studies against both embelin and a second GPR84 agonist, 6-(octylamino)-2,4(1H,3H)-pyrimidinedione (6-OAU)^2,8,9,11^ (Figure 1a). This provided a preliminary hit list (PHL) of 260 compounds (Figure 1b). Because some of the hits possessed chemical similarity to cAMP phosphodiesterase inhibitors, which would also be expected to elevate cAMP levels in intact cells but in a manner independent of GPR84, we further assessed the PHL compounds in an orthogonal assay using membrane preparations generated from Flp-In TREx 293 cells stably expressing a human GPR84-Gα_i2_ fusion protein.^17^ Here, we measured the ability of compounds to prevent stimulation of binding of [^35^S]GTPγS induced by embelin (Figures 1a, 1b). Following initial studies that were conducted at 3 μM (Figure 1b), concentration–response curves of 98 compounds that were confirmed as actives in this distinct assay resulted in a qualified hit list (QHL) of 49 compounds following triage based on potency and chemical characteristics (Figure 1a). Such studies identified compounds with IC_50_ (inhibitor concentration 50%) potency of <100 nM against an EC_80_ (effective concentration 80%) of embelin, potentially indicative of low nanomolar affinity at GPR84. Among these was the 1,2,4-triazine, designated compound 837, which was shown to have the highest potency. Synthesis of the anticipated molecule (3-((5,6-bis(4-methoxyphenyl)-1,2,4-triazin-3-yl)methyl)-1*H*-indole) (Figure 1c) confirmed its action as a high potency blocker of human GPR84.

### Pharmacological Characterization of GPR84 Antagonists

**Figure 2:**
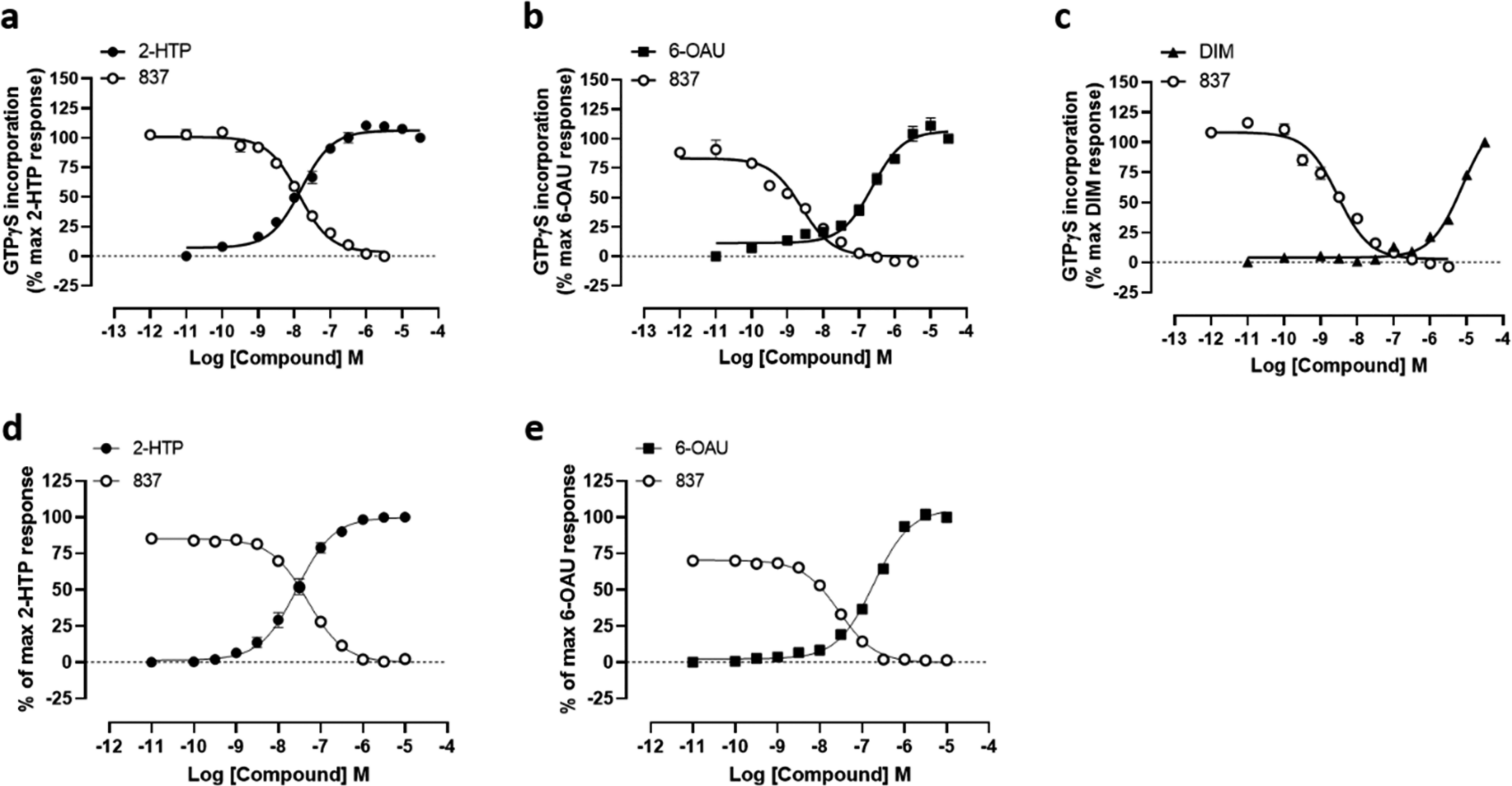
Compound 837 is a high potency antagonist of both orthosteric and allosteric activators of GPR84. Ability of varying concentrations of compound 837 to inhibit the orthosteric GPR84 agonists 2-HTP (a, d), 6-OAU (b, e), and the allosteric GPR84 activator DIM (c) was assessed in [^35^S]GTPγS binding (a–c) and cAMP (d, e) assays using Flp-In TREx 293 cells expressing the human GPR84-Gα_i2_ fusion protein (d, e) or membranes derived from these cells (a–c). Concentration–response data for each agonist are also displayed and used to assess EC_80_ concentrations for the studies with 837.

2-(Hexylthiol)pyrimidine-4,6 diol (2-HTP)^2,10^ is a recently described and significantly more potent agonist of GPR84 than either 6-OAU or embelin. Compound 837 was able, in a concentration-dependent manner, to fully block activation of human GPR84 promoted by each of 2-HTP (Figure 2a) and 6-OAU (Figure 2b) in [^35^S]GTPγS binding assays conducted in membranes of Flp-In TREx 293 cells expressing a human GPR84-Gα_i2_ fusion protein. Both 2-HTP and 6-OAU are considered orthosteric agonists of GPR84.^2,11^ By contrast, 3,3′-diindolylmethane (DIM) is an allosteric GPR84 activator^18^ that binds at a site distinct from 2-HTP, 6-OAU and other orthosteric agonists.^2,11,17,19^ Compound 837 also fully reversed activation of human GPR84 produced by DIM (Figure 2c). Moreover, compound 837 also fully blocked, in a concentration-dependent manner, inhibition of forskolin-amplified cAMP levels produced by both 2-HTP (Figure 2d) and 6-OAU (Figure 2e) in these same cells.

**Figure 3:**
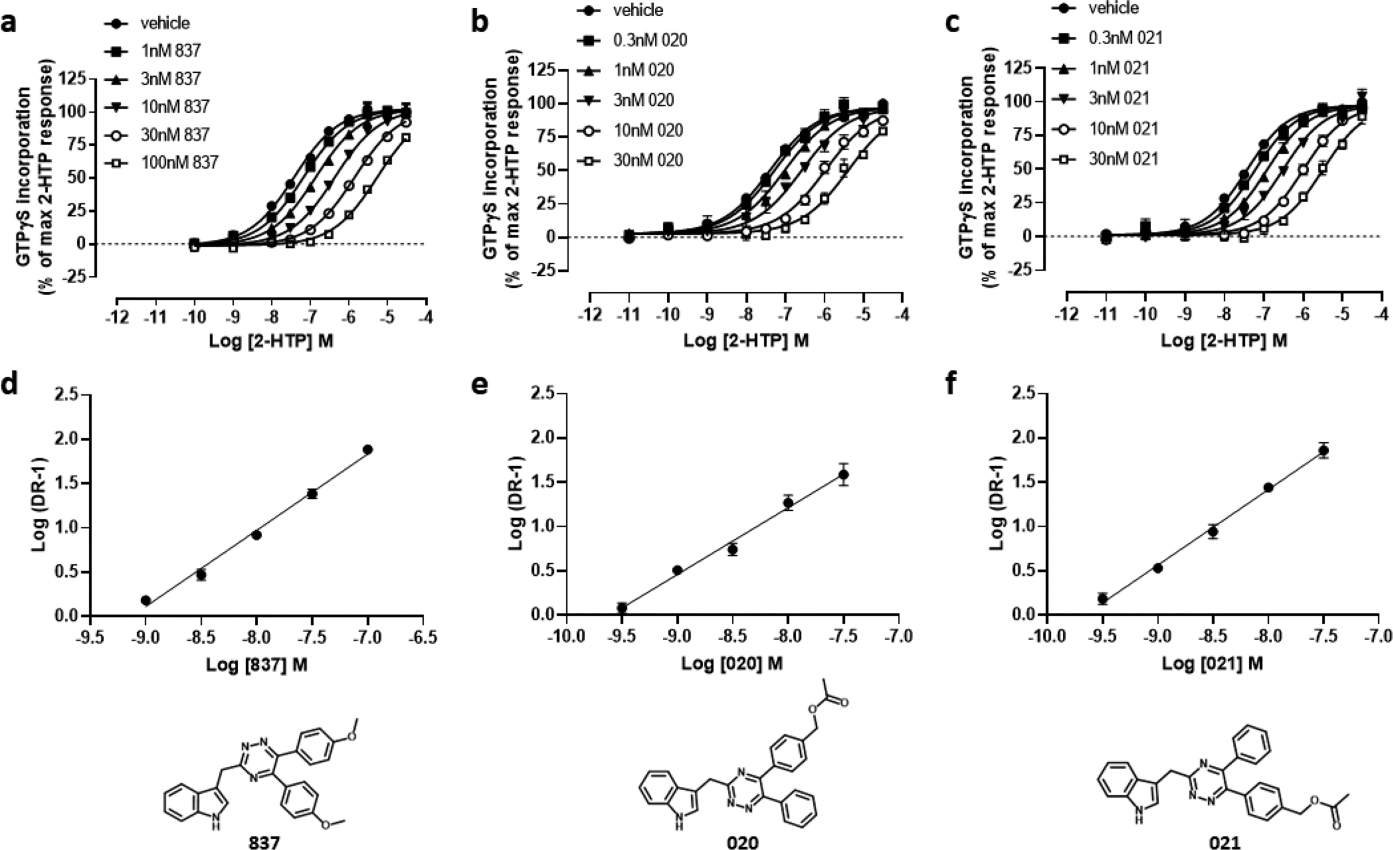
Compound 837 and more potent analogues are competitive orthosteric antagonists at human GPR84. Ability of varying concentrations of compound 837 (a), compound 020 (b), and compound 021 (c) (structures shown below) to alter the concentration–response curve of 2-HTP in [^35^S]GTPγS binding assays performed using membranes of Flp-In TREx 293 cells induced to stably express a human GPR84-Gα_i2_ fusion protein. (d–f) Schild plots of the data shown in panels a–c. Estimated Gaddum/Schild slopes from such plots (d, 1.11 ± 0.04; e, 1.27 ± 0.03; f, 1.14 ± 0.03) were not different from 1.0 (*p* > 0.05), consistent with a competitive mode of action.

Compound 837 acted as an orthosteric antagonist. Measured EC_50_ (effective concentration 50%) of 2-HTP was increased a concentration-dependent manner by compound 837 in [^35^S]GTPγS binding studies (Figure 3a), with estimated affinity of compound 837 (pA_2_) 8.90 ± 0.08 (mean ± SEM, *n* = 4), i.e. 1.26 nM. Compound 837 was also competitive with 2-HTP because the inhibitory effect of compound 837 was fully overcome upon addition of increasing concentrations of the agonist (Figure 3a). Limited chemistry around the structure of compound 837 generated compounds 020 (4-(3-((1*H*-indol-3-yl)methyl)-6-phenyl-1,2,4-triazin-5-yl)benzyl acetate) and 021 (4-(3-((1*H*-indol-3-yl)methyl)-5-phenyl-1,2,4-triazin-6-yl)benzyl acetate)that remained competitive with 2-HTP but displayed even higher affinity (020, pA_2_ = 9.19 ± 0.10, 021, pA_2_ = 9.31 ± 0.10, means ± SEM, *n* = 3) (Figures 3b, 3c).

### The Molecular Basis of Species Orthologue Selectivity

**Figure 4:**
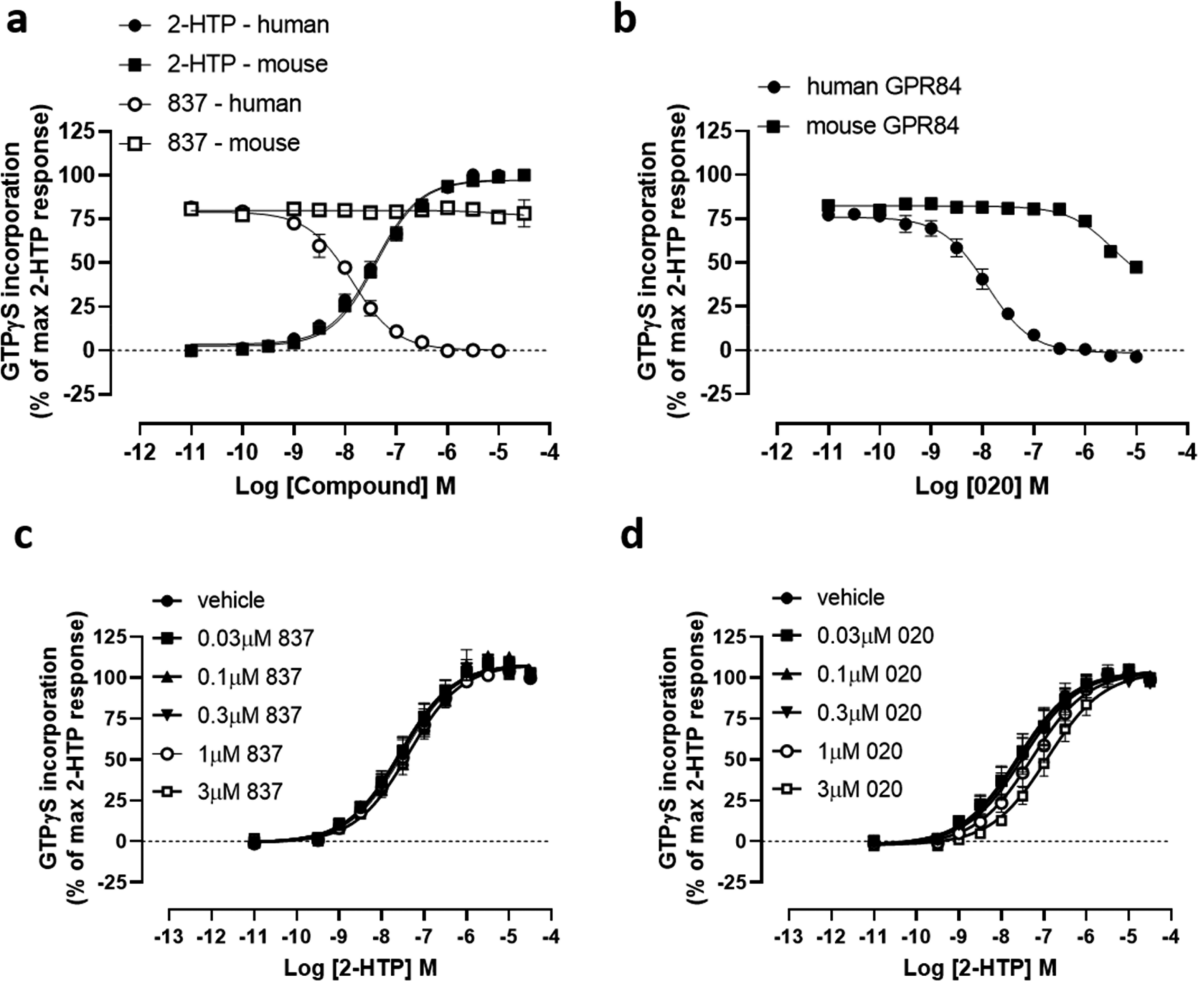
The 1,2,4-triazine ligands are not effective antagonists of mouse GPR84. Ability of increasing concentrations of compound 837 (a) and compound 020 (b) to antagonize effects of EC_80_ concentrations of 2-HTP at human (circles) and mouse (squares) GPR84 in [^35^S]GTPγS binding assays performed on membranes of Flp-In TREx 293 cells induced to stably express either a human or mouse GPR84-Gα_i2_ fusion protein is shown. The low affinity of 837 (c) and compound 020 (d) at mouse GPR84 was confirmed by their lack of ability to substantially effect the observed EC_50_ of 2-HTP at the mouse GPR84-Gα_i2_ fusion protein.

Mouse GPR84 displayed equal potency for the agonist 2-HTP (EC_50_ 7.38 ± 0.06, mean ± SEM, *n* = 3) as the human orthologue (EC_50_7.41 ± 0.10, mean ± SEM, *n* = 3). However, despite the high affinity of compound 837 for human GPR84 this ligand displayed no significant capacity to antagonize mouse GPR84 (Figure 4a), while compound 020 displayed only limited effects at concentrations at least 1000-fold higher than required to antagonize human GPR84 (Figure 4b). Indeed, using Gaddum/Schild analysis no measurable pA_2_ could be recorded for compound 837 (Figure 4c), while pA_2_ < 6.0 was estimated for compound 020 at mouse GPR84 (Figure 4d).

**Figure 5:**
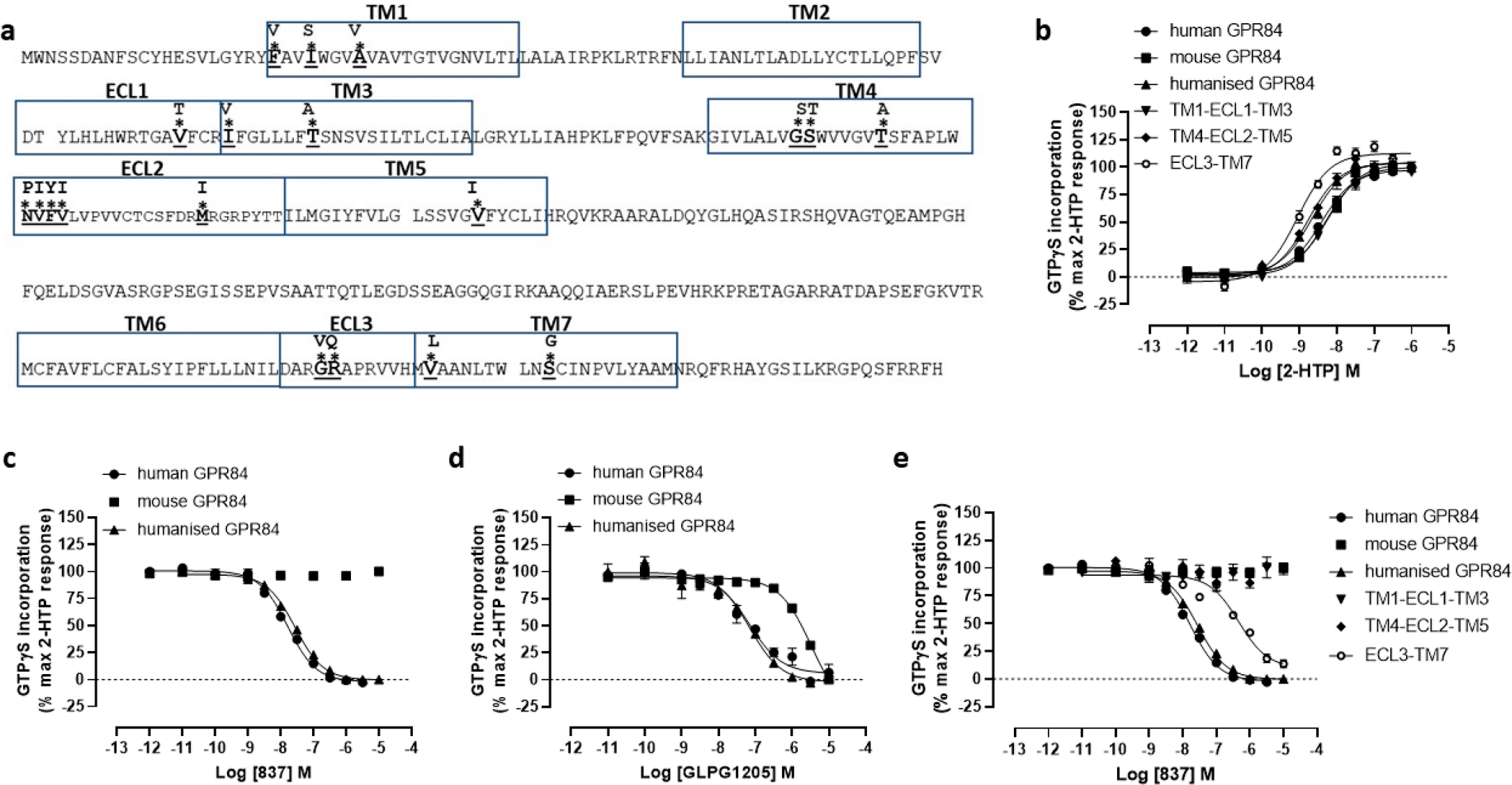
Chimeras of human/mouse GPR84 demonstrate lack of a linear segment responsible for the difference in antagonist affinity. Primary amino acid sequence of mouse GPR84 is shown with predicted extracellular regions and transmembrane domains boxed (a). Differences in these regions in human GPR84 are highlighted (*). A series of synthetic cDNA sequences able to encode chimeric human/mouse forms of GPR84 were generated and expressed stably in Flp-In TREx 293 cells. Humanized mGPR84 corresponds to the form in which all the differing residues were altered to the human residue. Each of the chimeras displayed similar potency of response to 2-HTP (b). 2-HTP-mediated activation of this “humanized” variant was antagonized by compound 837 as effectively as wild-type human GPR84 (c). This was also the case for GLPG1205, which is known to have lower affinity at mouse GPR84 compared to human^12^ (d). By contrast, synthetic chimeras that introduced various linear or discontinuous segments of human GPR84 into the mouse orthologue, with the exception of the ECL3/TM7 form, all failed to generate a form where the activity of 2-HTP was blocked by compound 837 (e).

Mouse and human GPR84 are some 85% identical in the extracellular (ECL) regions and transmembrane domains (TMDs) (Figure 5a). To attempt to define the basis for the species selectivity of compound 837, we synthesized a series of gene chimeras in which we introduced differences in these regions from the human receptor into the mouse orthologue. Following expression, each of these forms displayed similar potency for 2-HTP (Figure 5b). These included a form in which all residues in the ECLs and TMDs that differ between these orthologues were altered to the human sequence within the backbone of mouse GPR84 (Figure 5b). Compound 837 displayed equal affinity at this chimera as at the wild-type human receptor (Figure 5c), hence defining that the human-mouse difference in affinity for 837 and related compounds must by imbued by residue(s) within the ECLs and/or TMD regions. Interestingly, the only previously described group of high affinity GPR84 antagonists are known to have some 30–60 fold lower affinity for the mouse orthologue than for human GPR84.^12,17^ The affinity of the exemplar member of this series, GLPG1205,^17^ also was equal at the chimera in which all ECL and TMD residues were from the human sequence as at the wild-type human sequence (Figure 5d). Despite substantial efforts to define the basis of selectivity for compound 837 more clearly using a chimera approach, for example by altering all of extracellular loop 3 (ECL3) and transmembrane domain 7 (TMD7), that contains 4 amino acid differences, TMD1 + ECL1 + TMD3 (6 differences), or the segments TMD4 + ECL2 + TMD5 (9 differences), where each of these constructs displayed similar potency for 2-HTP (Figure 5b), none of these regional chimeras gained substantial affinity for compound 837 although the ECL3/TM7 gained some affinity (pIC_50_ = 6.44 ± 0.1) (Figure 5e).

**Figure 6:**
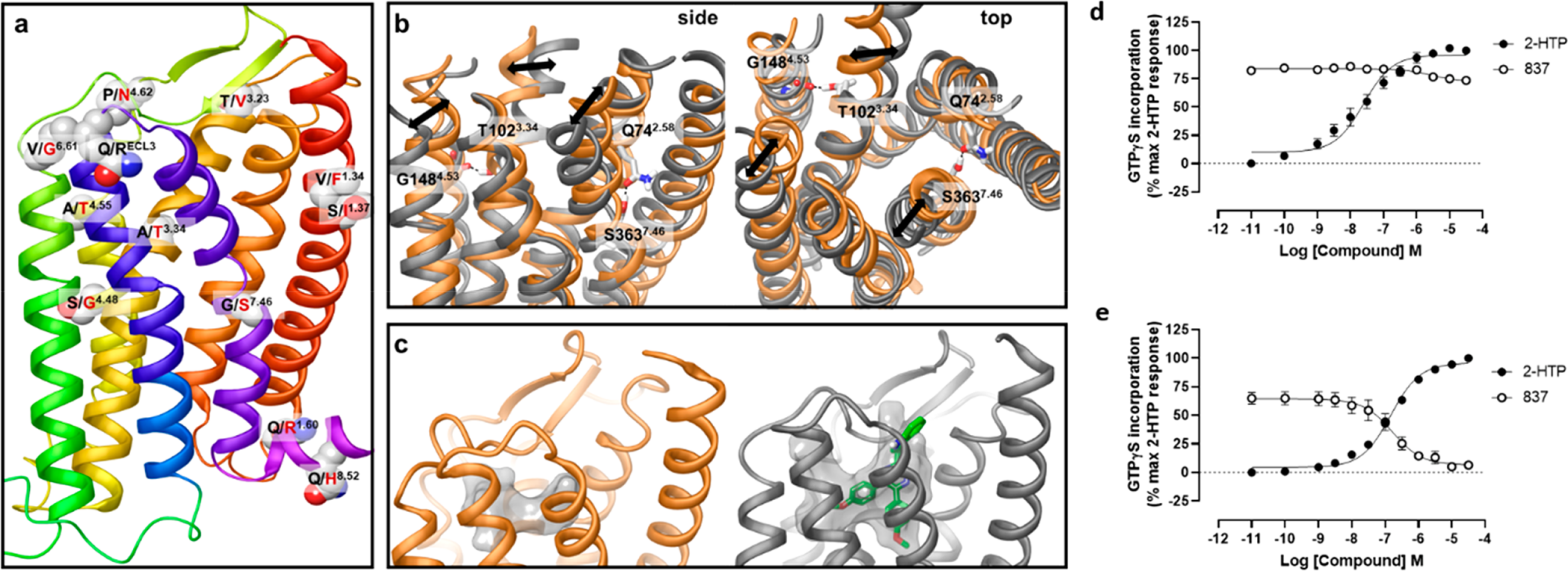
Dynamics of mouse and human GPR84 homology models helps to predict amino residues responsible for species selectivity of the 1,2,4-triazine antagonists. Nonconserved residues between mouse and human orthologues are shown on the human GPR84 homology model in space-filling representation (a). The receptor model is shown in cartoon representation. Nonconserved residues of the third intracellular loop are omitted. Amino acids from human and mouse are shown in black and red and residue labels respectively using Ballesteros–Weinstein residue location numbering. Overlay of the average mouse (orange) and human (gray) GPR84 structures from MD simulations shown from side and top (from the receptor extracellular opening) views (b). Nonconserved residues and their counterparts in interhelical hydrogen bonding are shown in stick-like representation. Hydrogen bonds are shown as black dashed lines. Black arrows show the difference in the position of helices 3, 5, and 7. The orthosteric binding cavity of mouse (orange) and human (gray) GPR84 detected by the MDpocket program^22^ from MD simulation trajectories shown in transparent surface representation (c). Human GPR84 is shown bound to compound 837 (green). Ala^102^Thr-Gly^363^Ser human GPR84-Gα_i2_ and Thr^102^Ala-Ser^363^Gly mouse GPR84-G_i2_α fusion proteins were produced and expressed stably in Flp-In TREx 293 cells (d, e). [^35^S]GTPγS binding assays performed on membranes of these cells showed that, although compound 837 was unable to effectively block activation of Ala^102^Thr-Gly^363^Ser human GPR84 by 2-HTP (d), it did effectively and fully block activation of Thr^102^Ala-Ser^363^Gly mouse GPR84 (e).

To explore this further, we turned to homology modeling and molecular dynamics (MD) simulations. In the absence of a GPR84 experimental structure or a 3D template of the receptor with high sequence similarity we built homology models of human and mouse GPR84 using a multitemplate hybridization approach. In this approach, a template of a TMD with the highest sequence similarity from GPCRs with available experimental structures was selected to model each TMD of GPR84. This approach provided a template with an average sequence similarity of 52% within the TMDs, and this was used to model human and mouse GPR84 structures. Once the TMD helices had been modeled, extracellular and intracellular loops were generated (see Methods), where ECL2 was modeled based on the rhodopsin template due to high sequence similarity.^20^ The location of nonconserved residues between mouse and human orthologues on the GPR84 homology model is shown in Figure 6a. To further establish the impact of these variations on the 3D structure, we conducted 300 ns MD simulations of the human and mouse GPR84 models in the empty form in a water–lipid bilayer. The GPR84 models were stable in the simulations with average carbon α atom root-mean-square deviation (RMSD) 3.0 ± 0.5A. From such MD simulations we assessed the position and interactions of the species nonconserved residues and noted that two of these, Thr^102^ (residue locator position 3.34^21^ and Ser^363^ (residue locator position 7.46) of the mouse receptor are engaged in interhelical hydrogen bonding. Specifically, Thr^102^ forms a hydrogen bond with Gly^148^ (residue locator position 4.53) while Ser^363^ interacts with Gln^74^ (residue locator position 2.58) (Figure 6b). Once formed these hydrogen bonds were maintained throughout the simulated time. The hydrogen bond between Ser^363^-Gln^74^ results in movement of the extracellular side of TMD helix 7 closer to helix 2, reducing the extracellular cavity involving helices 1, 2, and 7. In contrast, Gly at position 7.46 in human GPR84 increases mobility and causes slight outward movement of helix 7. We also observed changes in the position of helices 3 and 5. Figure 6c shows the average cavity in the extracellular side of the helical bundle of human and mouse GPR84 models from MD simulations detected by the MDpocket tool^22^ and the putative docking pose of compound 837 within human GPR84. As can be seen, the size and the shape of the binding cavities in mouse and human GPR84 are distinct, providing a potential structural basis as to why the compound series was not capable of binding to mouse GPR84. No other notable difference in interactions and conformations of other nonconserved residues were observed during the simulations. Such studies suggested that combined alteration (human to mouse) of Ala^102^Thr and Gly^363^Ser might be sufficient to limit or prevent binding of antagonists from this chemical series. As anticipated because, as highlighted above, in contrast to these antagonists the potency of agonist ligands is very similar at human and mouse GPR84, 2-HTP potently activated Ala^102^Thr, Gly^363^Ser human GPR84 (Figure 6d). However, compound 837 was now unable to block this effect (Figure 6d). To extend these studies, we performed the reverse mutations and generated Thr^102^Ala, Ser^363^Gly mouse GPR84. Now, although the EC_50_ of 2-HTP at this variant was reduced by some 3-fold compared to wild-type mouse GPR84, compound 837 was effective as an antagonist and able to fully block activation by 2-HTP (Figure 6e). To gain further insights, we assessed the affinity of compound 837 at both Thr^102^Ala, Ser^363^Gly mouse GPR84 and Ala^102^Thr, Gly^363^Ser human GPR84 by measuring the extent of displacement of the EC_50_ of 2-HTP to higher concentrations in the presence of increasing concentrations of compound 837 at both the residue swap mutants and the corresponding wild-type orthologues. The affinity of compound 837 at mouse GPR84 was immeasurably low (pA_2_ < 4.00, i.e. <100 μM) compared to human GPR84 (pA_2_ = 8.9, see above). However, at Thr^102^Ala, Ser^363^Gly mouse GPR84 the estimated pA_2_ of compound 837 was 7.78 ± 0.08 (mean ± SEM *n* = 3), only some 13-fold lower than at wild-type human GPR84 and at least 5000-fold higher than at wild-type mouse GPR84. Reciprocally, although Ala^102^Thr, Gly^363^Ser human GPR84 retained measurable affinity for compound 837 (pA_2_ = 6.33 ± 0.15, mean ± SEM *n* = 3) this was 380-fold lower than at wild-type human GPR84.

### Production and Studies with a [^3^H]antagonist

**Figure 7:**
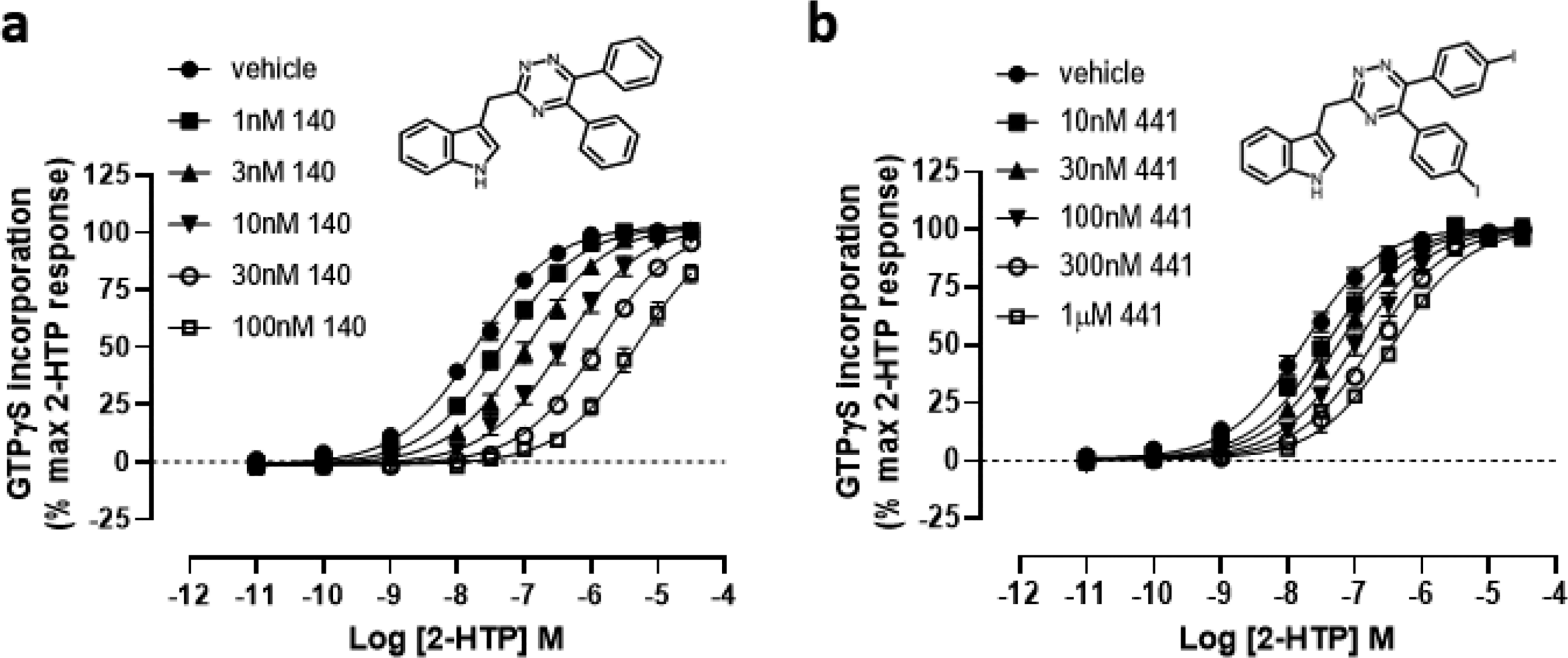
Characterization of compounds 140 and 441. Ability of varying concentrations of compound 140 (a) and compound 441 (b) (structures shown as inserts) to alter the concentration–response curve of 2-HTP in [^35^S]GTPγS binding assays performed using membranes of Flp-In TREx 293 cells induced to stably express a human GPR84-Gα_i2_ fusion protein. See Table 1 for details.

To further explore the binding characteristics of compounds from this series to forms of GPR84 we took advantage of a di-iodinated variant of the core structure. Although compound 441 (3-((5,6-bis(4-iodophenyl)-1,2,4-triazin-3-yl)methyl)-1*H*-indole) displayed reduced potency compared to certain other compounds (Table 1), we rationalized that tritiation of this compound would generate [^3^H]140, and compound 140 (3-((5,6-diphenyl-1,2,4-triazin-3-yl)methyl)-1*H*-indole) had already been characterized as a high affinity (pA_2_ = 9.13 ± 0.07, mean ± SEM, *n* = 3) competitive antagonist of human GPR84 (Figure 7 and Table 1). Moreover, submission of compound 140 to a DiscoverX panel of 167 GPCRs using the PathHunter β-arrestin enzyme fragment complementation (EFC) technology resulted in no significant capacity to either activate or antagonize any of these receptors (Supporting Information Table 1), indicating high selectivity for GPR84.

**Table 1:** Affinity of Compounds 140 and 441 at Human GPR84^a^

compound	pA_2_
441	8.07 ± 0.02
140	9.13 ± 0.07

a pA_2_ values were calculated by Gaddum/Schild analysis using shifts in the potency of 2-HTP induced by varying concentrations of compound 140 or 441. Data are presented as means ± SEM, *n* = 3.

**Figure 8:**
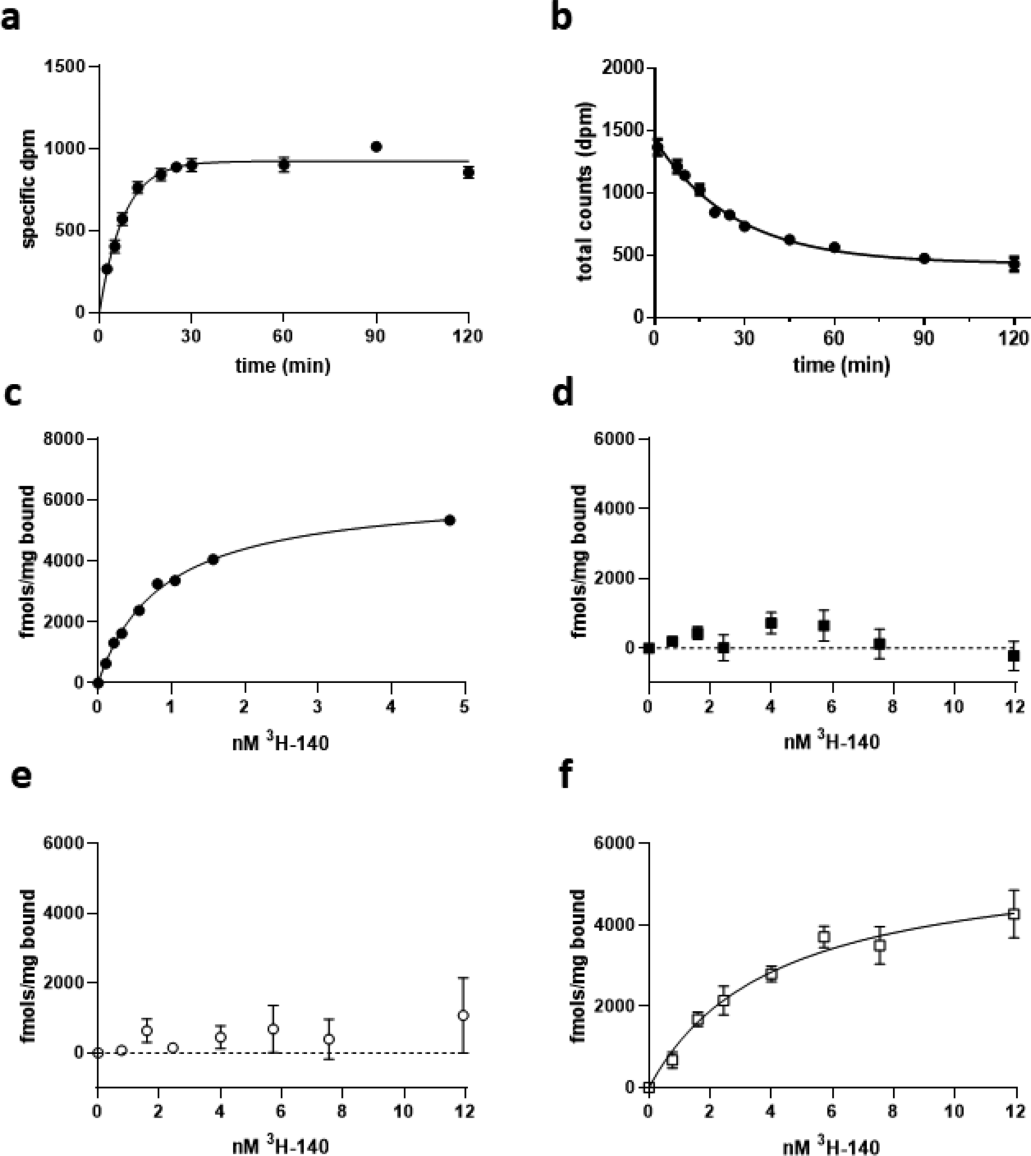
Characterization of [^3^H]140 binding. Association (a) and dissociation (b) kinetics of specific binding of 1.02 nM [^3^H]140 to membranes of Flp-In TREx 293 cells induced to express a human GPR84-Gα_i2_ fusion protein are shown from representative experiments. Dissociation was assessed in the presence of 1 μM compound 020 to prevent reassociation of the radioligand. Subsequently, the specific binding of a range of concentrations of [^3^H]140 to membranes of Flp-In TREx 293 cells induced to express the human GPR84-Gα_i2_ fusion protein was measured (c). Similar studies were performed on membranes expressing Ala^102^Thr-Gly^363^Ser human GPR84-Gα_i2_ (d), mouse GPR84-Gα_i2_ (e), and Thr^102^Ala-Ser^363^Gly mouse GPR84-Gα_i2_ (f). Specific binding with estimated *K*_d_ 4.7 ± 0.8 nM was observed to Thr^102^Ala-Ser^363^Gly mouse GPR84-Gα_i2_.

[^3^H]140 displayed rapid association (Figure 8a) and dissociation (Figure 8b) kinetics to membranes of Flp-In TREx 293 cells expressing a human GPR84-Gα_i2_ fusion protein, allowing an estimate of *K*_d_ as 0.4 nM. Good specific to nonspecific binding ratios were observed, and the specific binding component assessed using varying concentrations of [^3^H]140 also was also consistent with sub-nM affinity (*K*_d_ = 0.77 ± 0.10 nM, mean ± SEM, *n* = 4) (Figure 8c). There was no evidence of high affinity off-target binding sites in this cell line because no specific binding of [^3^H]140 was recorded in membranes from parental Flp-In TREx 293 cells. Unsurprisingly, in parallel studies we were unable to detect specific binding of [^3^H]140 in membranes of Flp-In TREx 293 cells stably transfected to express a mouse GPR84-Gα_i2_ fusion protein (Figure 8d). The greatly reduced affinity of compound 837 at Ala^102^Thr, Gly^363^Ser human GPR84 meant, however, that as anticipated, it was also not possible to measure binding of [^3^H]140 directly to this mutant (Figure 8e). By contrast, direct binding of [^3^H]140 to Thr^102^Ala, Ser^363^Gly mouse GPR84 was both easily measured and of high affinity (*K*_d_ = 4.7 ± 0.8 nM, mean ± SEM, *n* = 3) (Figure 8f).

### Further Predictions and Outcomes

**Figure 9:**
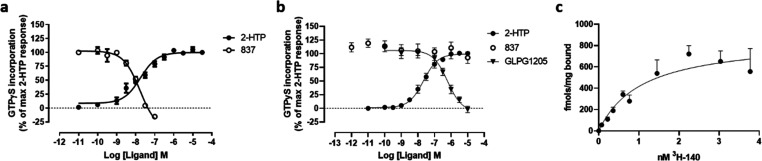
Human-derived THP-1 cells but not mouse-derived RAW-264.7 cells display responses to 2-HTP that are blocked by compound 837. Membranes were prepared from LPS-treated THP-1 cells and RAW-264.7 cells. In both cases, 2-HTPstimulated binding of [^35^S]GTPγS in a concentration-dependent manner (a, b). Only, however, in THP-1 cell membranes was this blocked by coaddition of compound 837. GLPG1205 was, however, able to block this effect of 2-HTPinRAW-264.7 cells (b). In THP-1 membranes, specific binding of [^3^H]140 was observed with *K*_d_ estimated as 1.3 ± 0.2 nM and *B*_max_ = 866 fmol/mg membrane protein (c).

Obvious sequalae of the species selectivity of compound 837 and related molecules were that while they should be effective blockers of function of GPR84 expressed endogenously in human-derived cell lines and tissue they should not act this way in mouse-derived cells and tissues. Two cell lines that have been well studied in relation to functions of GPR84 are the human monocytic/macrophage line THP-1^1,10,23^ and the murine macrophage-like line RAW264.7.^1,10^ Both show strong upregulation of GPR84 following exposure to lipopolysaccharide (LPS). In membranes derived from LPS-treated THP-1 cells, binding of [^35^S]GTPγS induced by 2-HTP was blocked in a concentration-dependent and high potency manner by compound 837 (Figure 9a). By contrast 837 was unable to produce such an effect in membranes of RAW264.7 stimulated with 2-HTP (Figure 9b). However, as anticipated GLPG1205 ref (12) was able to block response to 2-HTP in membranes of RAW264.7 cells (Figure 9b). Specific binding of [^3^H]140 in membranes of LPS-treated THP-1 cells was of high affinity (*K*_d_ = 1.3 ± 0.12 nM) with *B*_max_ = 905 ± 93 fmol/mg membrane protein (each mean ± SEM, *n* = 3) (Figure 9c).

**Figure 10:**
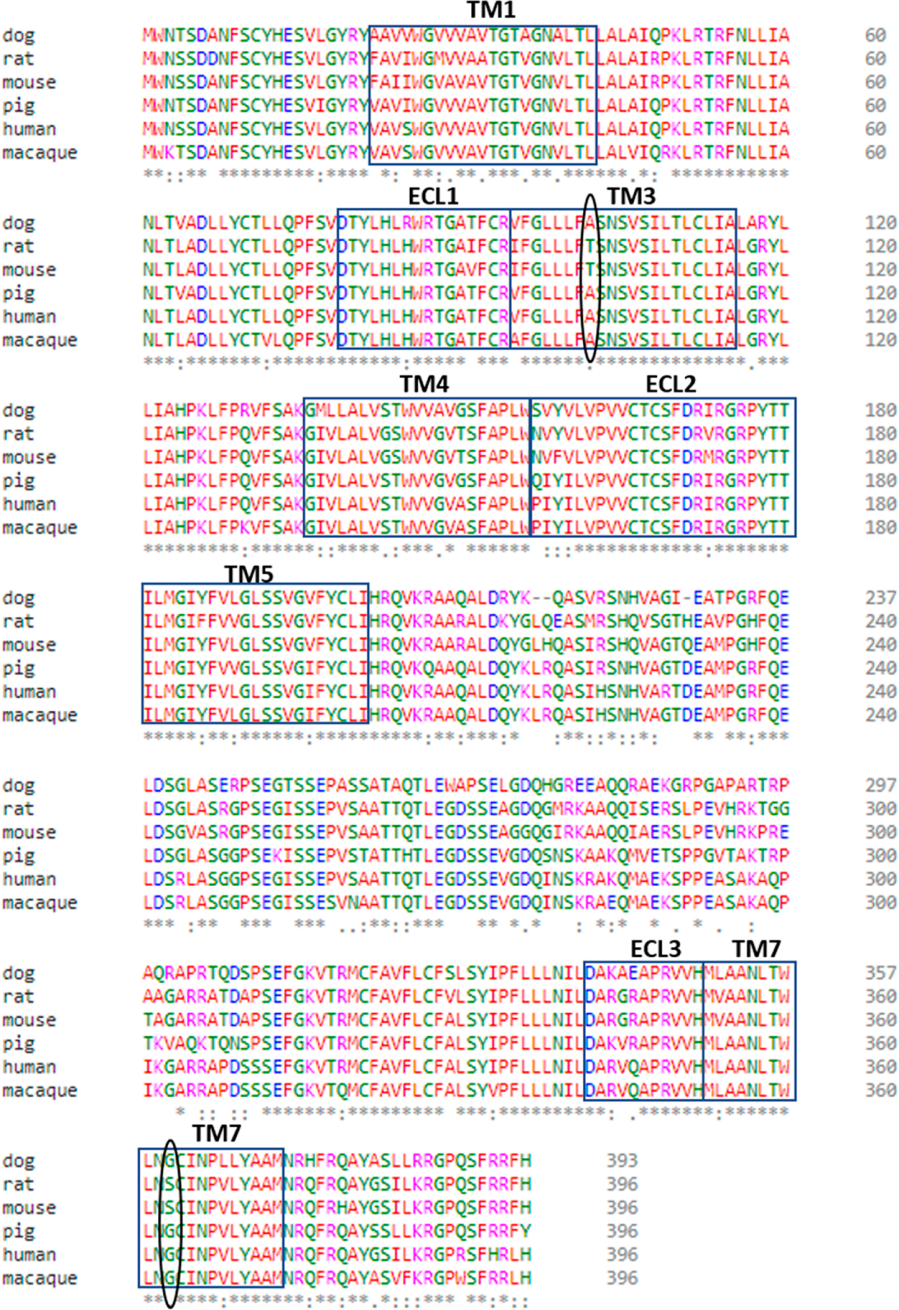
Alignment of species orthologues of GPR84 predicts those able to be blocked by the 1,2,4-triazines. GPR84 orthologues in human, mouse, rat, pig, dog, and macaque are aligned with residues at position indicator locations 3.34 and 7.46 highlighted. Based on the models of Figure 6, it was predicted that dog, pig, and macaque GPR84 would be antagonized effectively by the 1,2,4-trazine ligands, and direct assessment supported these predictions (Table 2).

Alignment of sequences of GPR84 from human, macaque, pig, dog, mouse, and rat indicated the amino acids at residue locations 3.34 and 7.46 were the same in macaque, pig, and dog as in human, and the same in rat as in mouse (Figure 10). A clear prediction was thus that compound 837 and related molecules from this series would be effective antagonists at each of macaque, pig, and dog GPR84, but not at the rat orthologue. This prediction was upheld when tested directly and with the measured affinity of such compounds being highly similar at the species orthologues predicted to be effective targets (Table 2).

**Table 2:** Potency of Compound 140 at Species Orthologues of GPR84^a^

compound 140	human	mouse	rat	dog	pig	macaque
IC_50_ (M)	6.0 × 10^–9^	>1 × 10^–5^	8.7 × 10^–6^	8.1 × 10^–9^	5.6 × 10^–9^	6.4 × 10^–9^

a IC_50_ versus EC_80_ of 6-OAU in cAMP regulation assays performed in CHO cells expressing the identified GPR84 species orthologue.

## Discussion

Although still classified as an orphan GPCR, GPR84 can be activated by medium chain length fatty acids, with C9–C11 chain length representing the peak of activity.^1,2,11^ A significant range of synthetic agonists with relatedness to fatty acids has been reported. These are hence considered as orthosteric agonists. In addition, initially, the single molecule DIM,^18^ and subsequently a substantial structure–activity analysis based on DIM,^19^ has defined a group of allosteric agonists.^2,11^ However, although there is interest in the potential of agonists of GPR84 as therapeutic agents,^2,11,16^ currently there is greater interest in the potential of antagonists of this receptor.^2,11^ Despite this interest and potential opportunity, the only high affinity series of GPR84 antagonists described previously is exemplified by GLPG1205.^12^ This ligand has been assessed clinically in both ulcerative colitis^12^ and in early studies in idiopathic pulmonary fibrosis.^13^ Although clinical efficacy end-points in ulcerative colitis were not achieved,^12^ the reported outcomes from the studies on idiopathic pulmonary fibrosis are encouraging (ClinicalTrials.gov Identifier: NCT03725852). A second molecule that has antagonist activity at GPR84 has also been studied in this condition,^24^ but PBI-4050^25,26^ and related molecules^27^ have very modest affinity at GPR84 and also have affinity at other GPCRs activated by fatty acids of varying chain length.^25^ It is thus clear that progress in this area would be promoted by the availability of distinct and selective high affinity antagonists of GPR84, and this was our rationale in attempts to uncover new ligands.

The current series was identified in a high throughput screen conducted in partnership with the European Lead Factory. Compound 837, with approximately 1 nM affinity at human GPR84, was identified directly from the more than 300 000 compounds assessed in the primary screen, which measured regulation of cAMP levels in CHO cells transfected to stably express the human receptor. Limited chemistry efforts identified even more high affinity ligands in compounds 140, 020, and 021, and a full description of the screen, medicinal chemistry and drug-like characteristics of these ligands will be reported elsewhere. Interrogation of the activity of these compounds in a broad panel screen, conducted externally, showed no significant activity against any of 167 different GPCRs, indicating the selectivity of this series for GPR84.

Although compound 837 and others from this series were high affinity antagonists of human GPR84, studies on the mouse orthologue indicated a virtual complete lack of activity. Although we have previously noted that available antagonists of receptors such as GPR35 are also highly selective for the human over mouse orthologue,^28^ orthologues of GPR35 are markedly different in primary sequence.^28^ By contrast, aside from the long third intracellular loop, orthologues of GPR84 display high similarity. Indeed, across the TMD domains and ECL regions, there is only modest variation. Because all agonists we have tested have very similar potency at the human and mouse orthologues we reasoned that it should be straightforward to generate functionally active chimeras between the species orthologues that would provide insight into both the location of binding of the antagonist series and the basis of species selectively. This, however, proved unsuccessful, although all the chimeras we did generate showed similar agonist potency responses following expression. Importantly, the basis for the selectivity clearly resided within the TMDs and/or ECL regions because when we replaced every residue in these areas of the mouse receptor with the equivalent amino acid from the human orthologue this modified form displayed fully “human orthologue-like” pharmacology.

To understand this species selectivity, we employed homology modeling and MD simulations. In previous work GPR84 homology models were generated based on each of the β_2_-adrenoceptor, dopamine D_3_ and orexin 2 receptors, that have average sequence similarities of 44, 45, and 47%, respectively, to GPR84.^20,29,30^ Here, we took advantage of recently published GPCR crystal structures and built novel homology models of GPR84 based on a hybrid template that gave overall sequence identity of 52% in the transmembrane regions. These models and their subsequent refinement with MD simulations helped us to predict two residues out of 12 nonconserved residues within the GPR84 TMDs and extracellular loops as providing the potential basis for species selectivity. Interactions involving the residues at amino acid locator positions 3.34 and 7.46 in mouse that did not exist in the human receptor were prioritized. MD simulations suggested these could restrict a cavity into which the antagonist might bind in the human orthologue. Generation of mutants in which we swapped these two amino acids between species provided strong support for this model. Introduction of the two residues from mouse into the human receptor resulted in reduction of more than 1000-fold in the affinity of exemplar ligands. Even more convincingly, introduction of these residues from the human receptor into the mouse orthologue resulted in a gain of affinity of some 5000-fold. An important consequence of these outcomes was that simple alignment of the sequences of orthologues of GPR84 from other species that are often important in pharmacological studies and drug-development programmes, including macaque, dog and rat, provided obvious predictions of the potential of high affinity binding or otherwise of the chemical series on these species. This reflected that rat was equivalent to mouse at residues 3.34 and 7.46, while macaque, dog and pig orthologues conserved these amino acids with human. Importantly, we then tested this prediction directly in cells transfected to express each of these species orthologues and the outcomes fully supported the model. Interestingly, although GLPG1205 and related molecules from that series do have substantial affinity at mouse GPR84, they display somewhere between 30- and 60-fold. lower affinity than at the human orthologue.^12,17^ As such, it appears that although the mode of binding of the GLPG1205 series compounds must differ from those reported herein, there may well be similarities. Direct structural insights into these probable differences would be welcome and helpful.

Although it is well established that mRNA encoding GPR84 is upregulated substantially and rapidly in cells and tissues in proinflammatory settings and in cells exposed to specific proinflammatory stimuli, including LPS,^1,9,10^ whether this results in a substantial and sustained increase in GPR84 protein expression has been less well assessed. This reflects the relative ease of PCR-based methods to detect and amplify mRNA and the relative paucity of antisera or other protein-targeted probes for the receptor. However, the availability of a [^3^H]radiolabeled ligand related closely to GLPG1205^12^ allowed Mancini et al.,^10^ to examine levels of expression of GPR84 protein in cells including THP-1 monocytes with and without pre-exposure to LPS.^10^ We hence generated [^3^H]140 in these studies, both to provide a chemically distinct radiolabeled probe for the type of studies outlined above and to allow more detailed pharmacological analysis of the binding site for these ligands in GPR84. This is currently being investigated directly.

In conclusion, from an initial high throughput screen we have identified, developed and characterized a completely novel series of high affinity GPR84 antagonists. Although these compounds display virtually complete selectivity between human and each of mouse and rat forms of GPR84, our analysis of sequence relatedness provides confidence over which species orthologues these compounds will interact with in a high affinity manner. They also highlight the minimal alterations that might be introduced, via genome-editing for example,^31^ to produce rodent models with nanomolar and subnanomolar affinity for these compounds. These ligands are also likely to offer suitable means to further assess the potential therapeutic targeting of GPR84 in a range of disease indications.

## Methods

### Materials

2,5-Dihydroxy-3-undecyl-1,4-benzoquinone (embelin), 3,3′-diindolylmethane (DIM), 6-*n*-octylaminouracil (6-OAU) and 2-(hexylthio)-6-hydroxy-4(3H)-pyrimidinone (2-HTP) were from Sigma-Aldrich (Dorset, UK). 9-cyclopropylethynyl-2-((*S*)-1-[1,4]dioxan-2-ylmethoxy)-6,7-dihydropyrimido[6,1-*a*]isoquinolin-4-one (GLPG1205)^12,17^ were kindly provided by Galapagos NV. [^35^S]GTPγS was from PerkinElmer Life Sciences (Beaconsfield, UK). Tissue culture reagents were from Thermo Fisher Scientific (Loughborough, UK) and molecular biology enzymes and reagents from Promega (Southampton, UK). Polyethylenimine (PEI) [linear poly(vinyl alcohol) (MW-25000)] was from Polysciences (Warrington, PA).

### Chemistry

#### Preparation of Compound 837, 3-((5,6-Bis(4-methoxyphenyl)-1,2,4-triazin-3-yl)methyl)-1*H*-indole

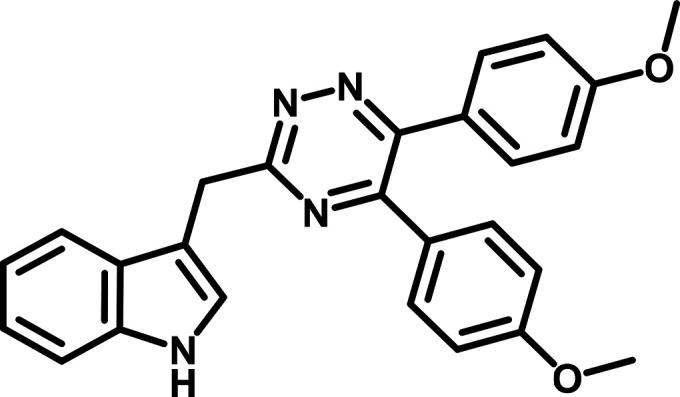



2-(1*H*-Indol-3-yl)acetohydrazide (100 mg, 0.58 mmol), 1,2-bis(4-methoxyphenyl)ethane-1,2-dione (156 mg, 0.58 mmol), and ammonium acetate (445 mg, 5.77 mmol) were transferred to a 5 mL microwave vial. Acetic acid (2.5 mL) was added and the resultant suspension subjected to microwave radiation at 180 °C for 5 min. The reaction mixture was concentrated to dryness under reduced pressure. Residue partitioned between water (20 mL) and DCM (2 × 10 mL). Combined extracts washed with brine, dried over Na_2_SO_4_, filtered, and concentrated to dryness under reduced pressure. The residue was purified by flash chromatography eluting with 0–2.5% methanol in DCM and then repurified by flash chromatography using 0–100% ethyl acetate in heptane to give a pale orange solid. The solid was sonicated with ether (10 mL) and solid filtered off, washed with ether and dried under suction to give the title compound, 47 mg.

^1^H NMR (400 MHz, CHLOROFORM-d) δ 3.85 (d, *J* = 1.25 Hz, 6Η), 4.70 (σ, 2Η), 6.79–6.95 (μ, 4Η), 7.12–7.26 (μ, 2Η), 7.33–7.42 (μ, 2Η), 7.47–7.54 (μ, 2Η), 7.55–7.63 (μ, 2Η), 7.90–8.01 (μ, 1Η), 8.07–8.21 (μ, 1Η).

#### Compound 140: Preparation of 3-((5,6-Diphenyl-1,2,4-triazin-3-yl)methyl)-1*H*-indole

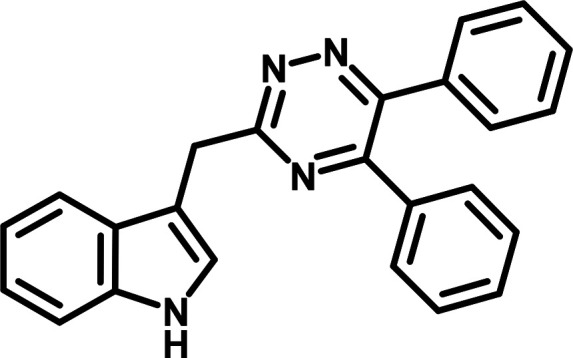



2-(1*H*-Indol-3-yl)acetohydrazide (100 mg, 0.58 mmol), 1,2-diphenylethane-1,2-dione (121. mg, 0.58 mmol) and ammonium acetate (445 mg, 5.77 mmol) transferred to a 5 mL microwave vial. Acetic acid (2.5 mL) was added and the resulting suspension subjected to microwave radiation at 180 °C for 5 min. The reaction mixture was concentrated to dryness under reduced pressure. Residue partitioned between water (20 mL) and DCM (2 × 10 mL) and combined extracts washed with brine, dried over Na_2_SO_4_, filtered, and concentrated to dryness under reduced pressure. The residue was purified by flash chromatography eluting with 0–30% ethyl acetate in heptane to give a pale orange solid. The solid was sonicated with ether (2 mL) and solid filtered off, washed with ether and dried under suction to give the title compound, 79.7 mg.

^1^H NMR (400 MHz, CHLOROFORM-d) δ 4.73 (d, *J* = 0.9 Hz, 2H), 7.20 (dddd, *J* = 20.4, 8.1, 7.1, 1.2 Hz, 2H), 7.30–7.47 (m, 8H), 7.54 (ddd, *J* = 8.3, 7.5, 1.5 Hz, 4H), 7.96 (dd, *J* = 7.5, 1.4 Hz, 1H), 8.16 (s, 1H).

#### Compound 441: Preparation of 3-((5,6-Bis(4-iodophenyl)-1,2,4-triazin-3-yl)methyl)-1*H*-indole

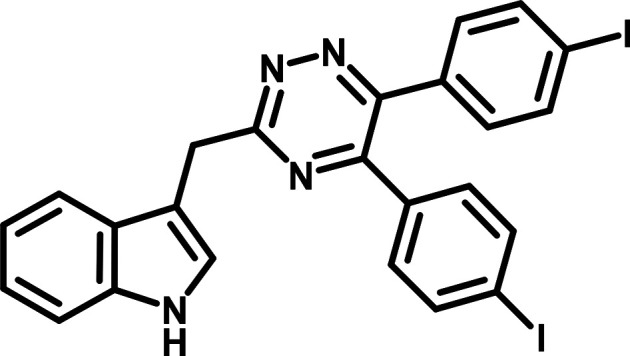



#### Preparation of 2-Hydroxy-1,2-bis(4-iodophenyl)ethan-1-one

To a stirred suspension of 4-iodobenzaldehyde (1000 mg, 4.31 mmol) in 1 mL of water/methanol (2:3) in a 5 mL microwave vial was added NaCN (42 mg, 0.862 mmol). The vial was capped and the mixture stirred at 85 °C for 30 min and cooled to room temperature. The reaction was worked up by partitioning mixture between water (20 mL) and ethyl acetate (3 × 10 mL). Combined extracts washed with brine (20 mL), dried over Na_2_SO_4_, filtered, and concentrated to dryness under reduced pressure. The resulting residue was purified by flash chromatography eluting with 0–20% ethyl acetate in heptane to give 2-hydroxy-1,2-bis(4-iodophenyl)ethan-1-one, 710 mg.

^1^Η ΝΜR (400 MHz, CHLOROFORM-d) δ 4.42–4.52 (m, 1Η), 5.81–5.90 (m, 1Η), 7.03–7.10 (m, 2Η), 7.56–7.63 (m, 2Η), 7.66–7.72 (m, 2Η), 7.78–7.84 (m, 2Η).

#### Preparation of 1,2-Bis(4-iodophenyl)ethane-1,2-dione

A well stirred suspension of 2-hydroxy-1,2-bis(4-iodophenyl)ethanone (710 mg, 1.53 mmol), ammonium nitrate (153 mg, 1.91 mmol) and copper(II) acetate monohydrate (3.05 mg, 0.0153 mmol) in AcOH (4.00 mL)/water (1.00 mL) was heated at reflux for 90 min resulting in a yellow suspension. The mixture cooled to room temperature and the resultant pale-yellow solid filtered off, washed with water (3 × 20 mL) and dried to give 1,2-bis(4-iodophenyl)ethane-1,2-dione, 563 mg.

^1^Η ΝΜR (400 MHz, CHLOROFORM-d) δ 7.67 (d, *J* = 8.53 Ηz, 4Η), 7.86–7.95 (m, 4Η).

#### H

2-(1-Indol-3-yl)acetohydrazide (231 mg, 1.22 mmol), 1,2-bis(4-iodophenyl)ethane-1,2-dione (563 mg, 1.22 mmol) and ammonium acetate (939 mg, 12.2 mmol) were transferred to a 5 mL microwave vial. Acetic acid (5 mL) was added and the resultant suspension subjected to microwave radiation at 180 °C for 5 min. The reaction mixture was concentrated to dryness under reduced pressure. The residue partitioned between water (30 mL) and DCM (3 × 20 mL) and combined extracts washed with brine, dried over Na_2_SO_4_, filtered, and concentrated to dryness under reduced pressure to afford a gum. The residue was purified by flash chromatography with 0–30% ethyl acetate in heptane to afford the title compound; 394 mg.

^1^Η ΝΜR (400 ΜΗz, DMSO-δ6) δ 4.51–4.59 (m, 2Η), 6.95–7.03 (m, 1Η), 7.03–7.10 (m, 1Η), 7.27 (s, 6Η), 7.65–7.73 (m, 1Η), 7.75–7.84 (m, 4Η), 10.89–11.03 (m, 1Η).

#### Compound 020 and Compound 021: Preparation of 4-(3-((1*H*-Indol-3-yl)methyl)-6-phenyl-1,2,4-triazin-5-yl)benzyl Acetate and 4-(3-((1*H*-Indol-3-yl)methyl)-5-phenyl-1,2,4-triazin-6-yl)benzyl Acetate

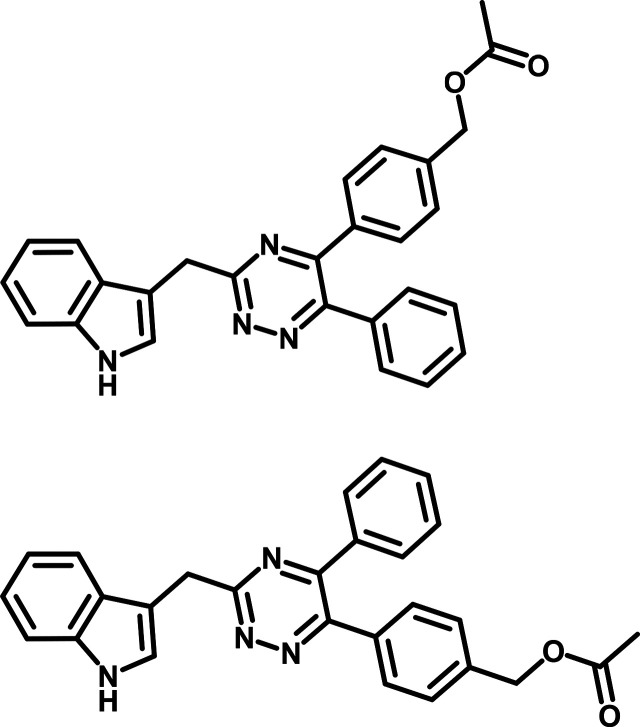



#### Preparation of [4-(2-Phenylethynyl)phenyl]methyl Acetate

(4-Bromophenyl)methyl acetate (960 mg, 4.19 mmol) and ethynylbenzene (460 μL, 4.19 mmol) were placed in a microwave vial and *N*,*N*-diisopropylethylamine (9 mL) added and the solution degassed by bubbling argon for 5 min. The cap was removed and copper(I) iodide (43 mg, 0.226 mmol) followed by bis(triphenylphosphine)palladium(II) dichloride (150 mg, 0.214 mmol) added and the vessel purged with argon before heating at 100 °C in the microwave for 1 h. The vial contents were diluted with ethyl acetate and passed through a Celite plug which was then washed with ethyl acetate. The organics were partitioned with water (2×) and sat. NaCl solution (1×), dried over Na_2_SO_4_, filtered, and concentrated. The resulting residue was purified by flash chromatography using 0–15% ethyl acetate in heptane to give [4-(2-phenylethynyl)phenyl]methyl acetate, 1.31 g contaminated with approximately 25% starting material. The material was used without further purification.

^1^H NMR (400 MHz, CHLOROFORM-d) δ 7.61–7.51 (m, 4H), 7.39–7.34 (m, 5H), 5.14 (s, 2H), 2.15 (s, 3H).

#### Preparation of [4-(2-Oxo-2-phenyl-acetyl)phenyl]methyl Acetate

[4-(2-phenylethynyl)phenyl]methyl acetate (75.0%, 1310 mg, 3.93 mmol) was dissolved in acetone (50 mL) and water (25 mL) added. Potassium permanganate (3102 mg, 19.6 mmol) was added, and the mixture stirred at room temperature for 1.5 h. The reaction mixture poured through a Celite pad which was washed with ethyl acetate and water. The filtrate was diluted with water (200 mL) and ethyl acetate (200 mL) and the phases mixed and separated. The organic layer separated and the aqueous back extracted with ethyl acetate (2 × 100 mL). The combined organics were washed with sat. NaCl solution, dried over Na_2_SO_4_, filtered, and concentrated to dryness. The residue was purified by flash chromatography eluting with 0–25% ethyl acetate in heptane to give [4-(2-oxo-2-phenyl-acetyl)phenyl]methyl acetate, 475 mg.

^1^H NMR (400 MHz, CHLOROFORM-d) δ 8.08–7.89 (m, 4H), 7.78–7.63 (m, 1H), 7.61–7.42 (m, 4H), 5.20 (s, 2H), 2.16 (s, 3H).

#### H

[4-(2-Oxo-2-phenyl-acetyl)phenyl]methyl acetate (475 mg, 1.68 mmol), 2-(1-indol-3-yl)acetohydrazide (0.353 mL, 1.68 mmol) and ammonium acetate (1.30 g, 16.8 mmol) were dissolved in 10 mL acetic acid and heated to 180 °C in the microwave for 10 min. The solution was concentrated to dryness and partitioned between ethyl acetate and sat. NaHCO_3_ and the phases mixed and separated. The aq. phase was back extracted with ethyl acetate and organics combined, dried over Na_2_SO_4_, filtered, and concentrated. The resulting residue was purified by flash chromatography using 0–50% ethyl acetate in heptane to give the regioisomeric mix. The regioisomers were separated by SFC using a Daicel AD-H (10 × 250 mm, 40% methanol, 15 mL/min) to give:

Compound 020, Daicel AD-H column 10 × 250 mm, 40% methanol, 15 mL/min, retention time 4.55 min, 102 mg.

^1^H NMP (400 ΜΗz, CHLOROFORM-d) δ 8.14–8.09 (m, 1Η), 7.97–7.94 (m, 1Η), 7.56–7.51 (m, 4Η), 7.45–7.29 (m, 7Η), 7.25–7.15 (m, 2Η), 5.13 (s, 2Η), 4.72–4.71 (m, 2Η), 2.14–2.14 (m, 3Η).

Compound 021, Daicel AD-H column 10 × 250 mm, 40% methanol, 15 mL/min, retention time 6.57 min, 99 mg.

^1^H NMR (400 ΜΗz, CHLOROFORM-d) δ 8.13–8.10 (m, 1Η), 7.97–7.94 (m, 1Η), 7.56–7.52 (m, 4Η), 7.47–7.32 (m, 7Η), 7.25–7.15 (m, 2Η), 5.15–5.14 (m, 2Η), 4.72 (s, 2Η), 2.14 (s, 3Η).

#### Synthesis of [^3^H]140

[^3^H]140 (3-((5,6-diphenyl-1,2,4-triazin-3-yl)methyl)-1*H*-indole) (40 Ci/mmol) was produced by Pharmaron (Cardiff, UK). It was synthesized by reaction of a solution of compound 441 (3-((5,6-bis(4-iodophenyl)-1,2,4-triazin-3-yl)methyl)-1*H*-indole) (5 mg) in *N*,*N*-dimethylformamide (1 mL) and diisopropylethylamine (0.02 mL) with tritium gas (5 Ci, 57.8 Ci/mmol) at 290 mbar pressure over 10% Pd/C (10 mg) for 2 h at room temperature. The labile activity was removed by rotary evaporation from ethanol three times before filtering through a 0.45 um GMF filter. The filtrate was purified by C18 RP HPLC eluting in a mixture of 0.1% TFA(aq) and MeCN. Concentration by rotary evaporation and then solvation in ethanol afforded the desired product as a solution (81 mCi) with 94.3% radiochemical purity by HPLC and an average molecular mass of 365.83 (MH+) by EI.

#### Generation of Constructs

FLAG-human GPR84-eYFP, FLAG-human GPR84-Gα_i2_, and FLAG-mouse GPR84-Gα_i2_ fusion proteins were constructed as described previously.^10,17^ An HA epitope (amino acid sequence YPYDVPDYA) was introduced at the C-terminal end of each of human and mouse GPR84 cDNA by PCR using the following primers: sense, 5′ GATCGATCGGATCCGCCACCATGTGGAACAGCTCAGATGCCAACTTCTCCTGCTACCATGAG 3′, and antisense: 5′ GATCGATCCTCGAGTTAATGGGTATGCTACAAGGTCTAATGCGAATGGAACCGGCGGAAACTCTGTGGCCCGCG 3′. The resulting cDNA was subsequently cloned in-frame into the *Bam*HI and XhoI sites of an pcDNA5/FRT/TO plasmid.

Chimeric human-mouse GPR84-HA constructs were generated using synthetic DNA sequences purchased from Eurofins Genomics (Luxembourg), that were then subcloned in-frame into the BamH1 and Xho1 sites of pcDNA5/FRT/TO.

#### Mutagenesis of FLAG-Human GPR84-Gα_i2_ and FLAG-Mouse GPR84-Gα_i2_

The Stratagene QuikChange method (Stratagene, Agilent Technologies, Santa Clara, CA) was used to introduce alterations into FLAG-human GPR84-Gα_i2_ or FLAG-mouse GPR84-Gα_i2_. Primers utilized for mutagenesis were provided by MWG Operon (Acton, UK). Template DNA was digested with DpnI to leave only the newly synthesized mutated plasmid, and sequencing was carried out to confirm the introduction of the alterations.

#### Cell Culture, Transfection, and Generation of Cell Lines

THP-1 monocytes were maintained at a density of between 1 × 10^5^– 8 × 10^5^ cells/mL in RPMI-1640 supplemented with 10% (v/v) heat inactivated FBS, 1% penicillin/streptomycin mixture and 2 mM l-glutamine at 37 °C in a 5% CO_2_ humidified atmosphere. Cells seeded at a density of 4 × 10^5^ cells/mL were exposed to 100 ng/mL lipopolysaccharide (LPS) (Sigma, Dorset, UK) for 24 h prior to membrane preparation. RAW 264.7 mouse macrophages were maintained in DMEM (with 4.5 g/L d-glucose, 0.11 g/L sodium pyruvate) supplemented with 10% heat inactivated FBS, 1% penicillin/streptomycin and 2 mM l-glutamine at 37 °C and 5% CO_2_ humidified atmosphere. Cells were treated with 100 ng/mL of LPS for 5 h prior to membrane preparation. Flp-In TREx 293 cells (Invitrogen) were maintained in Dulbecco’s modified Eagle’s medium without sodium pyruvate, supplemented with 10% (v/v) FBS, 1% penicillin/streptomycin mixture, and 10 μg/mL blasticidin at 37 °C in a 5% CO_2_ humidified atmosphere.

To generate Flp-In TREx 293 cells able to express in an inducible manner the various GPR84 receptor constructs, cells were transfected with a mixture containing the desired cDNA in pcDNA5/FRT/TO vector and pOG44 vector (1:9) by using 1 mg/mL PEI (MW-25000). Cells were plated until 60 to 80% confluent then transfected with 8 μg of required plasmid DNA and PEI (ratio 1:6 DNA/PEI), diluted in 150 mM NaCl, pH 7.4. After incubation at room temperature for 10 min, the mixture was added to cells. After 48 h, the medium was changed to medium supplemented with 200 μg/mL hygromycin B to initiate the selection of stably transfected cells. After isolation of resistant cells, expression of the appropriate construct from the Flp-In TREx locus was induced by treatment with up to 100 ng/mL doxycycline for 24 h.

#### HTRF-Based cAMP Inhibition Assays

cAMP experiments were performed using Flp-In T-REx293 cells induced to express the receptor of interest or CHO-K1 cells stably expressing the orthologue of interest. Experiments were carried out using a homogeneous time-resolved FRET-based detection kit (CisBio, Codolet, France) according to the manufacturer’s protocol. For the assay cells were plated at 5000 cells/well in low-volume 384-well plates. The ability of agonists to inhibit 1 μM forskolin-induced cAMP production was assessed following a preincubation for 15 min with antagonist compounds, then a further 30 min incubation with agonist compounds. Reactions were stopped according to the manufacturer’s instructions and the output was measured with a PHERAstar FS plate reader (BMG Labtech, Aylesbury, UK).

#### Membrane Preparation

Membranes were generated from LPS-treated THP-1 and RAW 264.7 cells or Flp-In T-Rex 293 cells following 100 ng/mL doxycycline treatment to induce receptor expression. Cells were washed with ice-cold phosphate-buffered saline (PBS), removed from dishes by scraping and centrifuged at 3000 rpm for 5 min at 4 °C. Pellets were resuspended in TE buffer (10 mM Tris-HCl, 0.1 mM EDTA; pH 7.5) containing a protease inhibitor mixture (Roche, West Sussex, UK) and homogenized with a 5 mL hand-held homogenizer. This material was centrifuged at 1500 rpm for 5 min at 4 °C and the supernatant was further centrifuged at 50 000 rpm for 45 min at 4 °C. The resulting pellet was resuspended in TE buffer and protein content was assessed using a BCA protein assay kit (Thermo Fisher Scientific, Loughborough, UK).

#### [^35^S]GTPγS Incorporation Assay

Prepared membrane protein (5 μg THP-1, 5 μg RAW-264.7, 3 μg Flp-In T-REx 293 cells) was incubated in assay buffer (20 mM HEPES, 5 mM MgCl_2_, 160 mM NaCl, 0.05% fatty-acid-free bovine serum albumin; pH 7.5) containing the indicated ligand concentrations. In experiments designed to assess inhibition of agonist stimulation, membrane preparations were preincubated with antagonist compound for 15 min at room temperature prior to addition of agonist. The reaction was initiated by addition of [^35^S]GTPγS (50 nCi per reaction) with 1 μM GDP, and incubated at 30 °C for 60 min. The reaction was terminated by rapid vacuum filtration through GF/C glass fiber filter-bottom 96-well microplates (PerkinElmer Life Sciences, Beaconsfield, UK) using a UniFilter FilterMate Harvester (PerkinElmer). Unbound radioligand was removed from filters by three washes with ice-cold PBS. MicroScint-20 (PerkinElmer) was added to dried filters, and [^35^S]GTPγS binding was quantified by liquid scintillation spectroscopy.

#### [^3^H]140 Binding Assay

Assays were carried out with increasing concentrations of [^3^H]140, binding buffer (PBS with 0.5% fatty acid free bovine serum albumin; pH 7.4), in a total assay volume of 500 μL in 96 deep-well blocks. Binding was initiated by the addition of membranes (5 μg of protein per tube). All assays were performed at 25 °C for 1 h before termination by the addition of ice-cold PBS and vacuum filtration through GF/C glassfibre filter-bottom 96-well microplates. Plates were washed three times with ice-cold PBS then allowed to dry for 2–3 h at room temperature. MicroScint-20 was added to dried filter plates, and radioactivity was quantified by liquid scintillation spectrometry. Specific binding was defined as the difference between binding detected in the presence and absence of 10 μM compound 020.

#### Studies on Species Orthologues of GPR84

Were performed on CHO-K1 cells stably expressing the GPR84 species orthologues described in the text.

#### Molecular Modeling

Homology models of human and mouse GPR84 were constructed using a multitemplate hybridization approach. The 3D model of GPR84 transmembrane helices was generated using the GPCR-SSFE 2.0 server.^32^ This server identifies templates for homology modeling based on key sequence and structural motifs of Class A GPCRs. The server suggested to use mOPRD1 (4EJ4), hDRD3 (3PBL), hFFAR1 (4PHU), hF2RL1 (5NDD), hS1PR1 (3 V2Y), hP2RY1 (4XNV), hF2RL1 (5NDD), hF2RL1 (5NDD) structures as templates for helices 1, 2, 3, 4, 5, 6, 7, and 8, respectively. The sequence similarity between the corresponding helices of GPR84 and the selected templates is 30, 57, 44, 46, 40, 53, 65, and 36%. Overall, the sequence similarity is higher for the hybrid template than for any GPCR with available experimental structures, suggesting that the hybrid template could be the best option for modeling GPR84 at this stage. Next, the homology model of the transmembrane helices was used to create the GPR84 models containing loop regions using the Prime module of Schrodinger software (2020–2021)^33^ with the default energy-based protocol. The second extracellular loop of GPR84 was modeled based on the rhodopsin template as it has high sequence similarity and similar sequence length.^20^ The third intracellular loop was partially reconstructed, where only ten residues from each connecting helix end were maintained. The obtained homology models of human and mouse GPR84 were subjected to MD simulations in a water–lipid bilayer for refinement and stability analysis.

The membrane-receptor systems were built using the “Membrane Builder” module of the CHARMM-GUI server.^34^ The position of the receptor molecule across the lipid bilayer was established using the Orientation of Protein in Membranes (OPM) server.^35^ Mouse or human GPR84 in the empty form was embedded into a 1-palmitoyl-2-oleoyl-*sn*-glycero-3-phosphocholine (POPC) lipid bilayer consisting of 103 and 100 lipid molecules in the upper-leaflet and lower-leaflet of the membrane. TIP3P water molecules^36^ were used to solvate the bilayer and counterions were added at a concentration of 0.15 M NaCl. The final systems comprised of ∼86 000 atoms with a box dimension of 90 × 90 × 114 Å^3^.

All MD simulations were performed using the Compute Unified Device Architecture (CUDA) version of particle-mesh Ewald molecular dynamics in Amber 18^37−39^ on graphics processing units (GPUs). The FF14SB^40^ and Lipid14^41^ force fields were used in all the simulations. The initial energy minimization of the entire systems used the steepest descent (5000 steps) and conjugate gradient (5000 steps) methods. The protein and bilayer were restrained using a potential of 10 and 2.5 kcal mol^–1^ Å^2^, respectively, and only solvent and ions were relaxed. Initial velocities were sampled from a Boltzmann distribution. Heating to 310 K was carried out in the NVT ensemble for a total of 125 ps. Equilibration was performed at 310 K and 1 bar in an NPT ensemble. During the equilibration, the restraints for the protein and lipid head groups were gradually reduced from 10 and 5 kcal mol^–1^ Å^–2^ for 125 ps; 5 and 2.5 kcal mol^–1^ Å^–2^ for 125 ps; 2.5 and 1.0 kcal mol^–1^ Å^–2^ for 125 ps; 1 and 0.5 kcal mol^–1^ Å^–2^ for 500 ps; 0.5 and 0.1 kcal mol^–1^ Å^–2^ for 500 ps; to 0.1 kcal mol^–1^ Å^–2^ (just for the protein) for 500 ps. The whole system was equilibrated without any restrains for 10 ns. The final production step of 300 ns was run at 310 K and 1 bar in the NPT ensemble using the Langevin thermostat and Monte Carlo barostat. The simulations were performed using a time step of 2 fs. Nonbonded interactions were cut off at 10.0 Å and long-range electrostatic interactions were calculated using PMEMD.^39,42^ The SHAKE algorithm was used to constrain bond lengths.^43^ Three replica runs of 300 ns MD simulation each were performed for all systems. The simulations were performed on the Kelvin2 cluster of Queen’s University Belfast. The MD trajectories were analyzed using the VMD 1.9.3^44^ and MDpocket programs.^22^ Compound 837 was docked to the human GPR84 model using a standard precision docking protocol available in the Glide module of Schrodinger software (2020–2021).^45^ The docking box was centered based on Asn104, Arg172 and Asn357 residues, which are known to be important in the binding of orthosteric ligands.^17,29^ The images for Figure 6 were created in Maestro 2020-1.

#### Primary Compound Screening

For primary screening a library of 301 665 compounds were tested in in a single-point HitHunter cAMP assay (DiscovereX, Fremont, CA), at 3 μM against 3 μM embelin in CHO-K1 cells expressing human GPR84. A primary hit list (PHL) of 260 compounds were selected and tested in a single-point [^35^S]GTPγS assay at 10 μM against 3 μM embelin. Basal wells containing assay buffer with 2.5% DMSO, and stimulation wells containing only 3 μM embelin were included in all plates. Data were analyzed using Microsoft Excel software, and the activity of the compounds was calculated using the following formula: % inhibition = (dpm_stim_ – dpm_compound_)/(dpm_stim_ – dpm_basal_) × 100, where dpm_compound_ is the dpm value obtained from wells treated with the test compound and embelin, dpm_basal_ is the average of the dpm values obtained from wells treated with assay buffer with 2.5% DMSO, and dpm_stim_ is the average of the dpm values obtained from cells treated with only 3 μM embelin. Reliability of the assay was estimated by calculating *Z*′ values for each plate, using the formula: *Z*′ = 1 – {[3 × σ_stim_) + (3 × σ_basal_)]/(μ_stim_ – μ_basal_)}, where σ_stim_ and σ_basal_ are the SD values of wells containing 3 μM embelin and assay buffer, respectively, and μ_stim_ and μ_basal_ are the means for wells containing 3 μM embelin and assay buffer, respectively.

#### Data Analysis

All data are presented as means ± SEM of at least three independent experiments. Data analysis and curve fitting was carried out using the GraphPad Prism software package version 8 (GraphPad, San Diego). For functional assays the concentration–response data were plotted on a log axis, with the untreated vehicle control plotted at 1 log unit lower than the lowest ligand concentration and fitted to a three parameter sigmoidal curve with the Hill slope constrained to equal 1. In case of inhibition experiments with antagonists, an equivalent analysis was followed to fit an inverse sigmoidal curve. To perform the statistical analysis of curve parameters, data from multiple experiments were fitted independently and resulting curve fit values were analyzed with indicated tests. Antagonism experiments carried out with multiple defined concentrations of antagonist were fit to a global Gaddum/Schild EC_50_ shift equation to estimate pA2 values for the antagonist. For radioligand binding data, saturation binding curves were generated by fitting the specific binding, which was obtained by subtracting nonspecific from total binding, to a one site specific binding model that allows calculation of *K*_d_ values for the radioligand at wild-type and mutant receptors.

## Supporting Information Available

Reference data (PDF)
