# Supplementary material for: Discovery and Characterization of Novel Antagonists of the Proinflammatory Orphan Receptor GPR84
Source: ACS Pharmacol Transl Sci. 2021 Sep 7;4(5):1598–613. doi: 10.1021/acsptsci.1c00151 (PMC8506611; doi:10.1021/acsptsci.1c00151)
Supplement: Supplementary file 1 — pt1c00151_si_001.pdf [file pt1c00151_si_001.pdf]

Supporting Information

Discovery and characterisation of novel antagonists of the pro-inflammatory orphan receptor GPR84

Laura Jenkins[1], Sara Marsango[1], Sarah Mancini[1], Zobaer Al Mahmud[1], Angus Morrison[2], Stuart P. McElroy[2], Kirstie A. Bennett[3], Matt Barnes[3], Andrew B. Tobin[1], Irina G. Tikhonova[4] and Graeme Milligan[1]*

1. The Centre for Translational Pharmacology, Institute of Molecular, Cellular and Systems Biology, College of Medical, Veterinary and Life Sciences, University of Glasgow, Glasgow G12 8QQ, United Kingdom
2. BioAscent Discovery Ltd., Bo'Ness Road, Newhouse, Lanarkshire, ML1 5UH, United Kingdom
3. Sosei Heptares, Steinmetz Building, Granta Park, Great Abington, Cambridge, CB21 6DG, United Kingdom
4. School of Pharmacy, Medical Biology Centre, Queen's University Belfast, Belfast, BT9 7BL, United Kingdom

***Address correspondence to Graeme Milligan (Graeme.Milligan@glasgow.ac.uk)**

Supporting Information

Details of the selectivity of compound 140 for GPR84 compared to 167 other human GPCRs

GPCR ID	Control 1	Mean RLU	SD	%CV	Control 2
ADCYAP1R1	Baseline	306530	48388	16%	Max
ADORA3	Baseline	281435	19159	7%	Max
ADRA1B	Baseline	763490	64542	8%	Max
ADRA2A	Baseline	442785	32399	7%	Max
ADRA2B	Baseline	198205	16825	8%	Max
ADRA2C	Baseline	193410	7216	4%	Max
ADRB1	Baseline	283850	23241	8%	Max
ADRB2	Baseline	30765	2010	7%	Max
AGTR1	Baseline	469105	51529	11%	Max
AGTRL1	Baseline	501620	13573	3%	Max
AVPR1A	Baseline	18375	2812	15%	Max
AVPR1B	Baseline	38955	2457	6%	Max
AVPR2	Baseline	705775	42501	6%	Max
BDKRB1	Baseline	17675	1937	11%	Max
BDKRB2	Baseline	581735	68687	12%	Max
BRS3	Baseline	185465	34954	19%	Max
C3AR1	Baseline	51275	3944	8%	Max
C5AR1	Baseline	131833	6869	5%	Max
C5L2	Baseline	488530	13746	3%	Max
CALCR	Baseline	69755	2616	4%	Max
CALCRL-RAMP1	Baseline	174615	18329	10%	Max
CALCRL-RAMP2	Baseline	1672230	66437	4%	Max
CALCRL-RAMP3	Baseline	52395	6426	12%	Max
CALCR-RAMP2	Baseline	68915	8007	12%	Max
CALCR-RAMP3	Baseline	41020	1625	4%	Max
CCKAR	Baseline	75355	8622	11%	Max
CCKBR	Baseline	1049195	99904	10%	Max
CCR10	Baseline	61670	8881	14%	Max
CCR1	Baseline	955710	19805	2%	Max
CCR2	Baseline	200270	8610	4%	Max
CCR3	Baseline	182210	18175	10%	Max
CCR4	Baseline	159507	7162	4%	Max
CCR5	Baseline	73535	7683	10%	Max
CCR6	Baseline	94150	10002	11%	Max
CCR7	Baseline	634410	40722	6%	Max
CCR8	Baseline	39200	3570	9%	Max
CCR9	Baseline	141330	11683	8%	Max
CHRM1	Baseline	509285	59486	12%	Max
CHRM2	Baseline	53760	10521	20%	Max
CHRM3	Baseline	58030	4814	8%	Max
CHRM4	Baseline	1844990	89513	5%	Max
CHRM5	Baseline	2612820	211446	8%	Max
CMKLR1	Baseline	73640	4265	6%	Max
CNR1	Baseline	63140	5915	9%	Max
CNR2	Baseline	322420	25065	8%	Max
CRHR1	Baseline	314860	20418	6%	Max
CRHR2	Baseline	91525	8927	10%	Max
CRTH2	Baseline	199115	27170	14%	Max
CX3CR1	Baseline	12600	1026	8%	Max
CXCR1	Baseline	56700	6231	11%	Max
CXCR2	Baseline	285880	12022	4%	Max

CXCR3	Baseline	387695	32807	8%	Max
CXCR4	Baseline	118230	5359	5%	Max
CXCR5	Baseline	162715	6697	4%	Max
CXCR6	Baseline	16520	1090	7%	Max
CXCR7	Baseline	314090	18891	6%	Max
DRD1	Baseline	205030	11406	6%	Max
DRD2L	Baseline	53795	6199	12%	Max
DRD2S	Baseline	179970	19333	11%	Max
DRD3	Baseline	347760	21125	6%	Max
DRD4	Baseline	11270	953	8%	Max
DRD5	Baseline	15085	1622	11%	Max
EBI2	Baseline	70595	7415	11%	Max
EDG1	Baseline	142520	9783	7%	Max
EDG3	Baseline	1370985	110553	8%	Max
EDG4	Baseline	229320	14785	6%	Max
EDG5	Baseline	208273	10288	5%	Max
EDG6	Baseline	243145	17426	7%	Max
EDG7	Baseline	267785	22751	8%	Max
EDNRA	Baseline	35070	1655	5%	Max
EDNRB	Baseline	116165	6389	6%	Max
F2R	Baseline	56980	11171	20%	Max
F2RL1	Baseline	366730	19421	5%	Max
F2RL3	Baseline	935445	61900	7%	Max
FFAR1	Baseline	158620	8248	5%	Max
FPR1	Baseline	1037960	76737	7%	Max
FPRL1	Baseline	136640	10558	8%	Max
FSHR	Baseline	109760	10673	10%	Max
GALR1	Baseline	791420	47463	6%	Max
GALR2	Baseline	242900	24816	10%	Max
GCGR	Baseline	263130	15582	6%	Max
GHSR	Baseline	708120	65523	9%	Max
GIPR	Baseline	14175	1905	13%	Max
GLP1R	Baseline	229740	14399	6%	Max
GLP2R	Baseline	101325	10115	10%	Max
GPR1	Baseline	75320	2739	4%	Max
GPR103	Baseline	65310	3588	5%	Max
GPR109A	Baseline	654570	85626	13%	Max
GPR109B	Baseline	577045	65480	11%	Max
GPR119	Baseline	256270	9606	4%	Max
GPR120	Baseline	98700	6539	7%	Max
GPR35	Baseline	378245	35447	9%	Max
GPR92	Baseline	142100	20174	14%	Max
GRPR	Baseline	28665	1443	5%	Max
HCRTR1	Baseline	173145	9951	6%	Max
HCRTR2	Baseline	63525	4103	6%	Max
HRH1	Baseline	183400	15746	9%	Max
HRH2	Baseline	82285	6382	8%	Max
HRH3	Baseline	68005	6906	10%	Max
HRH4	Baseline	459527	17711	4%	Max
HTR1A	Baseline	2166605	36509	2%	Max
HTR1B	Baseline	1692705	96418	6%	Max
HTR1E	Baseline	24430	1540	6%	Max

HTR1F	Baseline	365645	17367	5%	Max
HTR2A	Baseline	443135	24842	6%	Max
HTR2C	Baseline	579390	22146	4%	Max
HTR5A	Baseline	1284710	79566	6%	Max
KISS1R	Baseline	39200	2117	5%	Max
LHCGR	Baseline	30625	2660	9%	Max
LTB4R	Baseline	250250	18485	7%	Max
MC1R	Baseline	9380	1188	13%	Max
MC3R	Baseline	7420	686	9%	Max
MC4R	Baseline	32060	1577	5%	Max
MC5R	Baseline	94045	4929	5%	Max
MCHR1	Baseline	45150	8459	19%	Max
MCHR2	Baseline	68635	3398	5%	Max
MLNR	Baseline	130200	18469	14%	Max
MRGPRX1	Baseline	2375170	82079	3%	Max
MRGPRX2	Baseline	302540	13236	4%	Max
MTNR1A	Baseline	142065	9720	7%	Max
NMBR	Baseline	139825	9004	6%	Max
NMU1R	Baseline	62405	2818	5%	Max
NPBWR1	Baseline	61390	5821	9%	Max
NPBWR2	Baseline	173565	9812	6%	Max
NPFFR1	Baseline	77980	7184	9%	Max
NPSR1B	Baseline	38850	1921	5%	Max
NPY1R	Baseline	48685	6395	13%	Max
NPY2R	Baseline	166355	17131	10%	Max
NTSR1	Baseline	661570	105523	16%	Max
OPRD1	Baseline	74095	5433	7%	Max
OPRK1	Baseline	29435	4089	14%	Max
OPRL1	Baseline	132195	10281	8%	Max
OPRM1	Baseline	97755	4619	5%	Max
OXER1	Baseline	97650	5852	6%	Max
OXTR	Baseline	40775	5415	13%	Max
P2RY1	Baseline	214025	15761	7%	Max
P2RY11	Baseline	65695	5291	8%	Max
P2RY12	Baseline	173775	21776	13%	Max
P2RY2	Baseline	431270	34227	8%	Max
P2RY4	Baseline	485310	54365	11%	Max
P2RY6	Baseline	299180	48715	16%	Max
PPYR1	Baseline	40565	3294	8%	Max
PRLHR	Baseline	61110	5421	9%	Max
PROKR1	Baseline	65205	3028	5%	Max
PROKR2	Baseline	17185	1079	6%	Max
PTAFR	Baseline	965965	71651	7%	Max
PTGER2	Baseline	12145	1555	13%	Max
PTGER3	Baseline	479535	36753	8%	Max
PTGER4	Baseline	55300	5230	9%	Max
PTGFR	Baseline	6510	1127	17%	Max
PTGIR	Baseline	248920	27831	11%	Max
PTHR1	Baseline	60830	4779	8%	Max
PTHR2	Baseline	103845	7896	8%	Max
RXFP3	Baseline	78295	6631	8%	Max
SCTR	Baseline	479115	32510	7%	Max

SSTR1	Baseline	14980	1343	9%	Max
SSTR2	Baseline	8447	649	8%	Max
SSTR3	Baseline	145600	16604	11%	Max
SSTR5	Baseline	319830	27864	9%	Max
TACR1	Baseline	888265	89369	10%	Max
TACR2	Baseline	731745	47624	7%	Max
TACR3	Baseline	326410	24535	8%	Max
TBXA2R	Baseline	207655	9494	5%	Max
TRHR	Baseline	23450	3237	14%	Max
TSHR(L)	Baseline	5180	741	14%	Max
UTR2	Baseline	110460	6145	6%	Max
VIPR1	Baseline	444570	38311	9%	Max
VIPR2	Baseline	371175	38856	10%	Max
ADCYAP1R1	EC80	1613010	92659	6%	Basal
ADORA3	EC80	991270	25529	3%	Basal
ADRA1B	EC80	2926175	104997	4%	Basal
ADRA2A	EC80	1548050	91109	6%	Basal
ADRA2B	EC80	709870	29047	4%	Basal
ADRA2C	EC80	1031310	40758	4%	Basal
ADRB1	EC80	749245	48367	6%	Basal
ADRB2	EC80	573650	44229	8%	Basal
AGTR1	EC80	2740640	110285	4%	Basal
AGTRL1	EC80	2427215	203512	8%	Basal
AVPR1A	EC80	297920	20357	7%	Basal
AVPR1B	EC80	222250	14780	7%	Basal
AVPR2	EC80	2824325	91833	3%	Basal
BDKRB1	EC80	66640	1762	3%	Basal
BDKRB2	EC80	4539640	195156	4%	Basal
BRS3	EC80	1561490	80882	5%	Basal
C3AR1	EC80	1343895	71272	5%	Basal
C5AR1	EC80	1369865	78498	6%	Basal
C5L2	EC80	1332345	68925	5%	Basal
CALCR	EC80	401730	5063	1%	Basal
CALCRL-RAMP1	EC80	1593340	45535	3%	Basal
CALCRL-RAMP2	EC80	4872455	461945	9%	Basal
CALCRL-RAMP3	EC80	376530	3129	1%	Basal
CALCR-RAMP2	EC80	339745	14308	4%	Basal
CALCR-RAMP3	EC80	99073	3614	4%	Basal
CCKAR	EC80	1511440	20369	1%	Basal
CCKBR	EC80	4100670	149138	4%	Basal
CCR10	EC80	834995	31565	4%	Basal
CCR1	EC80	1596035	43075	3%	Basal
CCR2	EC80	1430380	64660	5%	Basal
CCR3	EC80	505610	7044	1%	Basal
CCR4	EC80	1139075	85658	8%	Basal
CCR5	EC80	1653540	97427	6%	Basal
CCR6	EC80	641445	19065	3%	Basal
CCR7	EC80	3230500	198592	6%	Basal
CCR8	EC80	1026480	50067	5%	Basal
CCR9	EC80	2216970	118574	5%	Basal
CHRM1	EC80	1420195	85785	6%	Basal
CHRM2	EC80	415800	38009	9%	Basal

CHRM3	EC80	364770	9380	3%	Basal
CHRM4	EC80	3244990	161581	5%	Basal
CHRM5	EC80	5782280	178339	3%	Basal
CMKLR1	EC80	2878155	163915	6%	Basal
CNR1	EC80	654640	19625	3%	Basal
CNR2	EC80	653427	33527	5%	Basal
CRHR1	EC80	4192125	154602	4%	Basal
CRHR2	EC80	1307985	34428	3%	Basal
CRTH2	EC80	855365	15529	2%	Basal
CX3CR1	EC80	160545	8942	6%	Basal
CXCR1	EC80	1585850	89774	6%	Basal
CXCR2	EC80	1010380	35447	4%	Basal
CXCR3	EC80	1268400	38591	3%	Basal
CXCR4	EC80	269430	20751	8%	Basal
CXCR5	EC80	1002120	38447	4%	Basal
CXCR6	EC80	45640	2483	5%	Basal
CXCR7	EC80	2936710	175856	6%	Basal
DRD1	EC80	1645770	66323	4%	Basal
DRD2L	EC80	196595	9288	5%	Basal
DRD2S	EC80	530110	34064	6%	Basal
DRD3	EC80	608755	15554	3%	Basal
DRD4	EC80	40530	2966	7%	Basal
DRD5	EC80	147910	9097	6%	Basal
EBI2	EC80	680890	95643	14%	Basal
EDG1	EC80	655130	32813	5%	Basal
EDG3	EC80	4585350	163045	4%	Basal
EDG4	EC80	482150	28251	6%	Basal
EDG5	EC80	1899660	149045	8%	Basal
EDG6	EC80	617330	27496	4%	Basal
EDG7	EC80	1596490	36588	2%	Basal
EDNRA	EC80	789740	14369	2%	Basal
EDNRB	EC80	2031995	139337	7%	Basal
F2R	EC80	276360	19255	7%	Basal
F2RL1	EC80	1466290	66717	5%	Basal
F2RL3	EC80	2804200	211734	8%	Basal
FFAR1	EC80	383390	19548	5%	Basal
FPR1	EC80	3728900	126482	3%	Basal
FPRL1	EC80	1172220	38464	3%	Basal
FSHR	EC80	566090	25708	5%	Basal
GALR1	EC80	4555460	73581	2%	Basal
GALR2	EC80	662795	41684	6%	Basal
GCGR	EC80	3201135	128252	4%	Basal
GHSR	EC80	1461355	40630	3%	Basal
GIPR	EC80	71890	2775	4%	Basal
GLP1R	EC80	689955	27659	4%	Basal
GLP2R	EC80	775250	38896	5%	Basal
GPR1	EC80	1450960	99327	7%	Basal
GPR103	EC80	155610	8816	6%	Basal
GPR109A	EC80	1869035	63181	3%	Basal
GPR109B	EC80	3370290	119637	4%	Basal
GPR119	EC80	494130	26012	5%	Basal
GPR120	EC80	262290	20061	8%	Basal

GPR35	EC80	864360	29295	3%	Basal
GPR92	EC80	674660	37672	6%	Basal
GRPR	EC80	725550	45540	6%	Basal
HCRTR1	EC80	2746065	121945	4%	Basal
HCRTR2	EC80	3119830	217792	7%	Basal
HRH1	EC80	994280	48090	5%	Basal
HRH2	EC80	372680	47809	13%	Basal
HRH3	EC80	150220	4649	3%	Basal
HRH4	EC80	1379910	71046	5%	Basal
HTR1A	EC80	4422390	40503	1%	Basal
HTR1B	EC80	3775415	138272	4%	Basal
HTR1E	EC80	74690	3808	5%	Basal
HTR1F	EC80	1131550	53043	5%	Basal
HTR2A	EC80	3392690	99734	3%	Basal
HTR2C	EC80	3015355	144541	5%	Basal
HTR5A	EC80	4004385	103096	3%	Basal
KISS1R	EC80	210945	10649	5%	Basal
LHCGR	EC80	222075	8637	4%	Basal
LTB4R	EC80	1594075	61328	4%	Basal
MC1R	EC80	31710	2040	6%	Basal
MC3R	EC80	43330	3074	7%	Basal
MC4R	EC80	218820	5652	3%	Basal
MC5R	EC80	281890	22950	8%	Basal
MCHR1	EC80	266980	6792	3%	Basal
MCHR2	EC80	560000	7803	1%	Basal
MLNR	EC80	1599710	57864	4%	Basal
MRGPRX1	EC80	5075595	246402	5%	Basal
MRGPRX2	EC80	1291115	83196	6%	Basal
MTNR1A	EC80	268520	9108	3%	Basal
NMBR	EC80	1722840	102801	6%	Basal
NMU1R	EC80	935060	76378	8%	Basal
NPBWR1	EC80	132580	4282	3%	Basal
NPBWR2	EC80	1399790	23208	2%	Basal
NPFFR1	EC80	182700	10256	6%	Basal
NPSR1B	EC80	356615	31665	9%	Basal
NPY1R	EC80	905555	48614	5%	Basal
NPY2R	EC80	3157175	177064	6%	Basal
NTSR1	EC80	2691080	129041	5%	Basal
OPRD1	EC80	993930	58880	6%	Basal
OPRK1	EC80	230650	3545	2%	Basal
OPRL1	EC80	468720	10933	2%	Basal
OPRM1	EC80	1462020	34976	2%	Basal
OXER1	EC80	280805	24501	9%	Basal
OXTR	EC80	564935	29679	5%	Basal
P2RY1	EC80	685685	18767	3%	Basal
P2RY11	EC80	418530	13472	3%	Basal
P2RY12	EC80	1007965	77831	8%	Basal
P2RY2	EC80	950915	51776	5%	Basal
P2RY4	EC80	1396080	35558	3%	Basal
P2RY6	EC80	977025	55233	6%	Basal
PPYR1	EC80	571060	11013	2%	Basal
PRLHR	EC80	268800	7576	3%	Basal

PROKR1	EC80	876120	18314	2%	Basal
PROKR2	EC80	347620	8370	2%	Basal
PTAFR	EC80	4197620	371764	9%	Basal
PTGER2	EC80	47355	4641	10%	Basal
PTGER3	EC80	1621410	54058	3%	Basal
PTGER4	EC80	1135190	72477	6%	Basal
PTGFR	EC80	186060	13673	7%	Basal
PTGIR	EC80	753830	16991	2%	Basal
PTHR1	EC80	2151660	112603	5%	Basal
PTHR2	EC80	2126460	68550	3%	Basal
RXFP3	EC80	202300	1154	1%	Basal
SCTR	EC80	2431485	80254	3%	Basal
SSTR1	EC80	58520	2452	4%	Basal
SSTR2	EC80	438655	40953	9%	Basal
SSTR3	EC80	480340	34766	7%	Basal
SSTR5	EC80	1263640	90682	7%	Basal
TACR1	EC80	5163550	188079	4%	Basal
TACR2	EC80	1522780	15006	1%	Basal
TACR3	EC80	1575350	29923	2%	Basal
TBXA2R	EC80	864010	52609	6%	Basal
TRHR	EC80	177170	14850	8%	Basal
TSHR(L)	EC80	49770	2718	5%	Basal
UTR2	EC80	499380	26222	5%	Basal
VIPR1	EC80	4351550	187802	4%	Basal
VIPR2	EC80	3586730	59019	2%	Basal

Mean RLU	SD	%CV	Assay Mode	Conc (µM)	Rep 1 RLU
1847020	20723	1%	Agonist	1	437080
1360520	23705	2%	Agonist	1	265720
4082470	104723	3%	Agonist	1	910000
1875230	5946	0%	Agonist	1	403760
1154300	37980	3%	Agonist	1	164360
1416800	43477	3%	Agonist	1	174160
926450	26960	3%	Agonist	1	275800
750190	26039	3%	Agonist	1	28840
3553410	49633	1%	Agonist	1	516880
3037930	56279	2%	Agonist	1	465640
414960	27740	7%	Agonist	1	15960
325430	7747	2%	Agonist	1	35280
3356640	68328	2%	Agonist	1	647360
94220	8537	9%	Agonist	1	17920
4877320	58614	1%	Agonist	1	470960
1641360	130839	8%	Agonist	1	207760
1832880	43159	2%	Agonist	1	50960
1551130	13353	1%	Agonist	1	117320
1412320	3368	0%	Agonist	1	431760
389200	14006	4%	Agonist	1	65520
2233560	90502	4%	Agonist	1	129640
5601820	146066	3%	Agonist	1	1434160
405090	13822	3%	Agonist	1	62160
461930	9741	2%	Agonist	1	73640
102060	7818	8%	Agonist	1	39200
2107980	22727	1%	Agonist	1	64680
4327750	46852	1%	Agonist	1	1127560
927360	65248	7%	Agonist	1	49840
1821470	51691	3%	Agonist	1	925680
1564010	19622	1%	Agonist	1	196280
554400	29042	5%	Agonist	1	168280
1553860	29840	2%	Agonist	1	197400
1785770	71486	4%	Agonist	1	90720
705810	51236	7%	Agonist	1	97720
3435600	169309	5%	Agonist	1	651000
1220030	50941	4%	Agonist	1	38920
1823547	196406	11%	Agonist	1	128240
1901550	91535	5%	Agonist	1	535920
701050	12844	2%	Agonist	1	58240
606270	15592	3%	Agonist	1	59360
3514840	18840	1%	Agonist	1	1678320
6031200	117165	2%	Agonist	1	2793000
3535490	102881	3%	Agonist	1	87920
1201970	53313	4%	Agonist	1	63840
691460	26836	4%	Agonist	1	361480
5079620	115416	2%	Agonist	1	358960
1729840	18692	1%	Agonist	1	97160
1214080	37000	3%	Agonist	1	164920
216580	4054	2%	Agonist	1	10640
1693440	26410	2%	Agonist	1	56280
1306830	17346	1%	Agonist	1	306320

1424710	60880	4%	Agonist	1	343000
331030	13158	4%	Agonist	1	135520
884940	109916	12%	Agonist	1	168560
59010	6279	11%	Agonist	1	18760
3195430	32719	1%	Agonist	1	325640
2075290	91829	4%	Agonist	1	199640
287630	5057	2%	Agonist	1	53760
590520	5014	1%	Agonist	1	193760
696430	8298	1%	Agonist	1	370160
48090	736	2%	Agonist	1	11760
197190	6204	3%	Agonist	1	15120
687960	70126	10%	Agonist	1	57960
951230	38148	4%	Agonist	1	166320
5539940	83855	2%	Agonist	1	1288280
618660	43209	7%	Agonist	1	170520
2165030	54674	3%	Agonist	1	210280
719250	72045	10%	Agonist	1	219240
1965600	24715	1%	Agonist	1	251160
800100	27234	3%	Agonist	1	31920
2388260	83468	3%	Agonist	1	117600
328230	20243	6%	Agonist	1	101920
1844640	49162	3%	Agonist	1	358400
2973110	111716	4%	Agonist	1	944160
435400	24224	6%	Agonist	1	161560
4330760	171966	4%	Agonist	1	1093400
1527190	37694	2%	Agonist	1	139440
656600	6677	1%	Agonist	1	120400
6061650	186749	3%	Agonist	1	780080
823270	35746	4%	Agonist	1	265720
3578120	105022	3%	Agonist	1	283360
1988700	22072	1%	Agonist	1	606480
86590	1606	2%	Agonist	1	13720
843570	27573	3%	Agonist	1	229320
1010730	8571	1%	Agonist	1	93520
1659070	70869	4%	Agonist	1	70000
183960	5679	3%	Agonist	1	60480
2188410	145771	7%	Agonist	1	617960
3562440	374256	11%	Agonist	1	578760
563080	9637	2%	Agonist	1	257040
364210	25836	7%	Agonist	1	84000
1350230	121065	9%	Agonist	1	413280
1013600	29431	3%	Agonist	1	126840
830760	17763	2%	Agonist	1	27160
3102190	109718	4%	Agonist	1	171920
3683190	47656	1%	Agonist	1	73080
1359890	48764	4%	Agonist	1	239400
509180	54156	11%	Agonist	1	87920
179130	7642	4%	Agonist	1	69720
1772050	66629	4%	Agonist	1	409360
5734050	128776	2%	Agonist	1	2098600
4741240	107351	2%	Agonist	1	1680840
93590	2137	2%	Agonist	1	26320

1410150	69864	5%	Agonist	1	398720
4240320	129237	3%	Agonist	1	489440
3823680	97468	3%	Agonist	1	567560
6054440	225431	4%	Agonist	1	1425480
283080	6336	2%	Agonist	1	37520
349580	42221	12%	Agonist	1	35280
2159780	140376	6%	Agonist	1	265160
37940	1793	5%	Agonist	1	9520
51030	3545	7%	Agonist	1	6160
295540	12635	4%	Agonist	1	35560
365610	16472	5%	Agonist	1	87080
345730	41432	12%	Agonist	1	34720
673890	14042	2%	Agonist	1	68600
2014250	18717	1%	Agonist	1	141120
5230400	158640	3%	Agonist	1	2419760
1895460	163412	9%	Agonist	1	356440
328790	9850	3%	Agonist	1	142240
1852270	73858	4%	Agonist	1	144480
1156680	9678	1%	Agonist	1	70000
170450	2125	1%	Agonist	1	64120
1539720	29970	2%	Agonist	1	185920
221900	4677	2%	Agonist	1	82880
394730	28360	7%	Agonist	1	47600
1139320	24650	2%	Agonist	1	45920
3876880	55443	1%	Agonist	1	199360
2931670	45014	2%	Agonist	1	623000
1208690	29580	2%	Agonist	1	77000
250110	7455	3%	Agonist	1	24360
697690	12266	2%	Agonist	1	112560
1815800	73587	4%	Agonist	1	96600
367360	33658	9%	Agonist	1	85120
783090	38788	5%	Agonist	1	45920
856520	20236	2%	Agonist	1	200200
304173	27125	9%	Agonist	1	57120
1339730	41985	3%	Agonist	1	169400
1127000	25266	2%	Agonist	1	431760
1853250	48584	3%	Agonist	1	464520
1351840	20574	2%	Agonist	1	265440
711550	16858	2%	Agonist	1	38640
311640	6839	2%	Agonist	1	71960
1124340	96140	9%	Agonist	1	76160
445410	48526	11%	Agonist	1	19040
5468820	72735	1%	Agonist	1	831880
67760	1600	2%	Agonist	1	9800
2059540	21140	1%	Agonist	1	492520
1208830	28108	2%	Agonist	1	65240
286020	11836	4%	Agonist	1	7280
1182440	27263	2%	Agonist	1	250600
2651180	36259	1%	Agonist	1	69160
2455460	47376	2%	Agonist	1	113680
271600	9894	4%	Agonist	1	69160
2887710	130080	5%	Agonist	1	537880

77700	10450	13%	Agonist	1	11760
507010	24820	5%	Agonist	1	7280
505260	33973	7%	Agonist	1	163520
1366680	36009	3%	Agonist	1	268520
5361230	140202	3%	Agonist	1	794920
2081310	153051	7%	Agonist	1	760480
2042740	21246	1%	Agonist	1	364840
1234380	17569	1%	Agonist	1	199640
202020	10092	5%	Agonist	1	22680
61600	3110	5%	Agonist	1	5320
618380	12085	2%	Agonist	1	108360
4884460	34237	1%	Agonist	1	467880
3958010	54621	1%	Agonist	1	386960
306530	48388	16%	Antagonist	1	1596280
281435	19159	7%	Antagonist	1	853440
763490	64542	8%	Antagonist	1	2544360
442785	32399	7%	Antagonist	1	1663760
198205	16825	8%	Antagonist	1	672280
193410	7216	4%	Antagonist	1	1009400
283850	23241	8%	Antagonist	1	761320
30765	2010	7%	Antagonist	1	543480
469105	51529	11%	Antagonist	1	2868600
501620	13573	3%	Antagonist	1	2114840
18375	2812	15%	Antagonist	1	244720
38955	2457	6%	Antagonist	1	186200
705775	42501	6%	Antagonist	1	2636480
17675	1937	11%	Antagonist	1	61040
581735	68687	12%	Antagonist	1	4441640
185465	34954	19%	Antagonist	1	1443960
51275	3944	8%	Antagonist	1	1446200
131833	6869	5%	Antagonist	1	1328320
488530	13746	3%	Antagonist	1	1242080
69755	2616	4%	Antagonist	1	401240
174615	18329	10%	Antagonist	1	1502480
1672230	66437	4%	Antagonist	1	4592840
52395	6426	12%	Antagonist	1	369040
68915	8007	12%	Antagonist	1	291760
41020	1625	4%	Antagonist	1	82320
75355	8622	11%	Antagonist	1	1547840
1049195	99904	10%	Antagonist	1	4100600
61670	8881	14%	Antagonist	1	804440
955710	19805	2%	Antagonist	1	1465800
200270	8610	4%	Antagonist	1	1468600
182210	18175	10%	Antagonist	1	504840
159507	7162	4%	Antagonist	1	1088640
73535	7683	10%	Antagonist	1	1650040
94150	10002	11%	Antagonist	1	616000
634410	40722	6%	Antagonist	1	3052840
39200	3570	9%	Antagonist	1	988680
141330	11683	8%	Antagonist	1	2011520
509285	59486	12%	Antagonist	1	1404200
53760	10521	20%	Antagonist	1	391720

58030	4814	8%	Antagonist	1	331240
1844990	89513	5%	Antagonist	1	2975000
2612820	211446	8%	Antagonist	1	5608120
73640	4265	6%	Antagonist	1	2965760
63140	5915	9%	Antagonist	1	571760
322420	25065	8%	Antagonist	1	564200
314860	20418	6%	Antagonist	1	3957520
91525	8927	10%	Antagonist	1	1358840
199115	27170	14%	Antagonist	1	742840
12600	1026	8%	Antagonist	1	145600
56700	6231	11%	Antagonist	1	1631280
285880	12022	4%	Antagonist	1	997640
387695	32807	8%	Antagonist	1	1216040
118230	5359	5%	Antagonist	1	245840
162715	6697	4%	Antagonist	1	1022000
16520	1090	7%	Antagonist	1	41160
314090	18891	6%	Antagonist	1	2843120
205030	11406	6%	Antagonist	1	1550360
53795	6199	12%	Antagonist	1	183400
179970	19333	11%	Antagonist	1	475160
347760	21125	6%	Antagonist	1	594720
11270	953	8%	Antagonist	1	42560
15085	1622	11%	Antagonist	1	138600
70595	7415	11%	Antagonist	1	519400
142520	9783	7%	Antagonist	1	654080
1370985	110553	8%	Antagonist	1	4382840
229320	14785	6%	Antagonist	1	430640
208273	10288	5%	Antagonist	1	1689800
243145	17426	7%	Antagonist	1	557200
267785	22751	8%	Antagonist	1	1338400
35070	1655	5%	Antagonist	1	713160
116165	6389	6%	Antagonist	1	1783880
56980	11171	20%	Antagonist	1	284200
366730	19421	5%	Antagonist	1	1509480
935445	61900	7%	Antagonist	1	2603160
158620	8248	5%	Antagonist	1	368480
1037960	76737	7%	Antagonist	1	3472560
136640	10558	8%	Antagonist	1	1085560
109760	10673	10%	Antagonist	1	440720
791420	47463	6%	Antagonist	1	4147920
242900	24816	10%	Antagonist	1	633920
263130	15582	6%	Antagonist	1	3060400
708120	65523	9%	Antagonist	1	1275680
14175	1905	13%	Antagonist	1	63840
229740	14399	6%	Antagonist	1	714840
101325	10115	10%	Antagonist	1	723240
75320	2739	4%	Antagonist	1	1432760
65310	3588	5%	Antagonist	1	134680
654570	85626	13%	Antagonist	1	1789200
577045	65480	11%	Antagonist	1	3353560
256270	9606	4%	Antagonist	1	443240
98700	6539	7%	Antagonist	1	259560

378245	35447	9%	Antagonist	1	832160
142100	20174	14%	Antagonist	1	544320
28665	1443	5%	Antagonist	1	738920
173145	9951	6%	Antagonist	1	2520560
63525	4103	6%	Antagonist	1	3085040
183400	15746	9%	Antagonist	1	998200
82285	6382	8%	Antagonist	1	337120
68005	6906	10%	Antagonist	1	140560
459527	17711	4%	Antagonist	1	1312920
2166605	36509	2%	Antagonist	1	4123840
1692705	96418	6%	Antagonist	1	3519320
24430	1540	6%	Antagonist	1	71120
365645	17367	5%	Antagonist	1	973280
443135	24842	6%	Antagonist	1	3227000
579390	22146	4%	Antagonist	1	2907800
1284710	79566	6%	Antagonist	1	4153520
39200	2117	5%	Antagonist	1	220080
30625	2660	9%	Antagonist	1	220920
250250	18485	7%	Antagonist	1	1456280
9380	1188	13%	Antagonist	1	32200
7420	686	9%	Antagonist	1	44240
32060	1577	5%	Antagonist	1	230160
94045	4929	5%	Antagonist	1	291760
45150	8459	19%	Antagonist	1	265720
68635	3398	5%	Antagonist	1	559160
130200	18469	14%	Antagonist	1	1584240
2375170	82079	3%	Antagonist	1	5062400
302540	13236	4%	Antagonist	1	1275680
142065	9720	7%	Antagonist	1	268520
139825	9004	6%	Antagonist	1	1835120
62405	2818	5%	Antagonist	1	900200
61390	5821	9%	Antagonist	1	132160
173565	9812	6%	Antagonist	1	1390480
77980	7184	9%	Antagonist	1	187600
38850	1921	5%	Antagonist	1	351400
48685	6395	13%	Antagonist	1	854840
166355	17131	10%	Antagonist	1	2934680
661570	105523	16%	Antagonist	1	2637320
74095	5433	7%	Antagonist	1	993160
29435	4089	14%	Antagonist	1	214200
132195	10281	8%	Antagonist	1	396760
97755	4619	5%	Antagonist	1	1477840
97650	5852	6%	Antagonist	1	294560
40775	5415	13%	Antagonist	1	498400
214025	15761	7%	Antagonist	1	697480
65695	5291	8%	Antagonist	1	420280
173775	21776	13%	Antagonist	1	861280
431270	34227	8%	Antagonist	1	988680
485310	54365	11%	Antagonist	1	1268960
299180	48715	16%	Antagonist	1	969640
40565	3294	8%	Antagonist	1	616280
61110	5421	9%	Antagonist	1	294560

65205	3028	5%	Antagonist	1	854000
17185	1079	6%	Antagonist	1	362880
965965	71651	7%	Antagonist	1	3724560
12145	1555	13%	Antagonist	1	44240
479535	36753	8%	Antagonist	1	1647520
55300	5230	9%	Antagonist	1	1140440
6510	1127	17%	Antagonist	1	175280
248920	27831	11%	Antagonist	1	600320
60830	4779	8%	Antagonist	1	2051560
103845	7896	8%	Antagonist	1	2255120
78295	6631	8%	Antagonist	1	184800
479115	32510	7%	Antagonist	1	2439080
14980	1343	9%	Antagonist	1	54320
8447	649	8%	Antagonist	1	398160
145600	16604	11%	Antagonist	1	468720
319830	27864	9%	Antagonist	1	1146040
888265	89369	10%	Antagonist	1	4827760
731745	47624	7%	Antagonist	1	1359680
326410	24535	8%	Antagonist	1	1483720
207655	9494	5%	Antagonist	1	810600
23450	3237	14%	Antagonist	1	187600
5180	741	14%	Antagonist	1	44800
110460	6145	6%	Antagonist	1	411320
444570	38311	9%	Antagonist	1	4007360
371175	38856	10%	Antagonist	1	3501120

Rep 2 RLU	Mean RLU	SD	%CV	% Activity
357000	397040	56625	14%	6%
268800	267260	2178	1%	-1%
878360	894180	22373	3%	4%
394800	399280	6336	2%	-3%
181720	173040	12275	7%	-3%
159320	166740	10493	6%	-2%
289240	282520	9504	3%	0%
31920	30380	2178	7%	0%
538440	527660	15245	3%	2%
482720	474180	12077	3%	-1%
15960	15960	0	0%	-1%
36680	35980	990	3%	-1%
634480	640920	9108	1%	-2%
20440	19180	1782	9%	2%
481320	476140	7326	2%	-2%
174160	190960	23759	12%	0%
56000	53480	3564	7%	0%
120120	118720	1980	2%	-1%
459480	445620	19601	4%	-5%
68040	66780	1782	3%	-1%
162960	146300	23561	16%	-1%
1359120	1396640	53061	4%	-7%
58520	60340	2574	4%	2%
75320	74480	1188	2%	1%
37520	38360	1188	3%	-4%
68040	66360	2376	4%	0%
1093400	1110480	24155	2%	2%
52080	50960	1584	3%	-1%
922040	923860	2574	0%	-4%
230720	213500	24353	11%	1%
172480	170380	2970	2%	-3%
174720	186060	16037	9%	2%
82600	86660	5742	7%	1%
103600	100660	4158	4%	1%
672840	661920	15443	2%	1%
36400	37660	1782	5%	0%
123200	125720	3564	3%	-1%
546280	541100	7326	1%	2%
55440	56840	1980	3%	0%
66920	63140	5346	8%	1%
1730680	1704500	37024	2%	-8%
2836680	2814840	30886	1%	6%
78960	83440	6336	8%	0%
59920	61880	2772	4%	0%
356720	359100	3366	1%	10%
347200	353080	8316	2%	1%
88480	92820	6138	7%	0%
164920	164920	0	0%	-3%
12040	11340	990	9%	-1%
54040	55160	1584	3%	0%
308000	307160	1188	0%	2%

360640	351820	12473	4%	-3%
133560	134540	1386	1%	8%
164640	166600	2772	2%	1%
19600	19180	594	3%	6%
310240	317940	10889	3%	0%
216720	208180	12077	6%	0%
58800	56280	3564	6%	1%
183960	188860	6930	4%	2%
359800	364980	7326	2%	5%
12040	11900	198	2%	2%
13720	14420	990	7%	0%
53480	55720	3168	6%	-2%
140560	153440	18215	12%	1%
1337560	1312920	34846	3%	-1%
222600	196560	36826	19%	-8%
210840	210560	396	0%	0%
225120	222180	4158	2%	-4%
234640	242900	11681	5%	-1%
35280	33600	2376	7%	0%
123760	120680	4356	4%	0%
109280	105600	5204	5%	18%
379680	369040	15047	4%	0%
937160	940660	4950	1%	0%
155400	158480	4356	3%	0%
1099560	1096480	4356	0%	2%
144200	141820	3366	2%	0%
112840	116620	5346	5%	1%
807520	793800	19403	2%	0%
271040	268380	3762	1%	4%
286440	284900	2178	1%	1%
571760	589120	24551	4%	-9%
16240	14980	1782	12%	1%
236040	232680	4752	2%	0%
87920	90720	3960	4%	-1%
72240	71120	1584	2%	0%
61040	60760	396	1%	-4%
642320	630140	17225	3%	-2%
588840	583800	7128	1%	0%
256200	256620	594	0%	0%
86240	85120	1584	2%	-5%
396480	404880	11879	3%	3%
126840	126840	0	0%	-2%
27720	27440	396	1%	0%
175560	173740	2574	1%	0%
65520	69300	5346	8%	0%
191240	215320	34054	16%	3%
71680	79800	11483	14%	-1%
66640	68180	2178	3%	0%
436240	422800	19007	4%	-3%
2108960	2103780	7326	0%	-2%
1709680	1695260	20393	1%	0%
26320	26320	0	0%	3%

362880	380800	25343	7%	1%
449960	469700	27917	6%	1%
578480	573020	7722	1%	0%
1374240	1399860	36232	3%	2%
32480	35000	3564	10%	-2%
38360	36820	2178	6%	2%
301000	283080	25343	9%	2%
8680	9100	594	7%	-1%
6160	6160	0	0%	-3%
31360	33460	2970	9%	1%
93520	90300	4554	5%	-1%
35000	34860	198	1%	-3%
68880	68740	198	0%	0%
140560	140840	396	0%	1%
2523080	2471420	73058	3%	3%
321720	339080	24551	7%	2%
136920	139580	3762	3%	-1%
122080	133280	15839	12%	0%
65240	67620	3366	5%	0%
56840	60480	5148	9%	-1%
181160	183540	3366	2%	1%
81480	82180	990	1%	3%
42840	45220	3366	7%	2%
50680	48300	3366	7%	0%
208040	203700	6138	3%	1%
607600	615300	10889	2%	-2%
79800	78400	1980	3%	0%
25760	25060	990	4%	-2%
119000	115780	4554	4%	-3%
90160	93380	4554	5%	0%
95480	90300	7326	8%	-3%
40040	42980	4158	10%	0%
207760	203980	5346	3%	-2%
64960	61040	5544	9%	-2%
173040	171220	2574	2%	0%
445480	438620	9702	2%	1%
489160	476840	17423	4%	-1%
232400	248920	23363	9%	-5%
40600	39620	1386	3%	0%
77280	74620	3762	5%	5%
83440	79800	5148	6%	1%
21280	20160	1584	8%	1%
853160	842520	15047	2%	-3%
11200	10500	990	9%	-3%
530320	511420	26729	5%	2%
68040	66640	1980	3%	1%
7000	7140	198	3%	0%
267120	258860	11681	5%	1%
67760	68460	990	1%	0%
108640	111160	3564	3%	0%
69160	69160	0	0%	-5%
489160	513520	34450	7%	1%

13440	12600	1188	9%	-4%
8400	7840	792	10%	0%
165200	164360	1188	1%	5%
274120	271320	3960	1%	-5%
815640	805280	14651	2%	-2%
776440	768460	11285	1%	3%
308000	336420	40192	12%	1%
221200	210420	15245	7%	0%
26040	24360	2376	10%	1%
4760	5040	396	8%	0%
117320	112840	6336	6%	0%
462000	464940	4158	1%	0%
388080	387520	792	0%	0%
1640520	1618400	31282	2%	0%
899080	876260	32272	4%	16%
2595040	2569700	35836	1%	16%
1601040	1632400	44350	3%	-8%
688520	680400	11483	2%	6%
1032920	1021160	16631	2%	1%
777560	769440	11483	1%	-4%
558600	551040	10691	2%	4%
2910600	2889600	29698	1%	-7%
2194360	2154600	56229	3%	14%
228480	236600	11483	5%	22%
197400	191800	7920	4%	17%
2701440	2668960	45934	2%	7%
53480	57260	5346	9%	19%
4358200	4399920	59001	1%	4%
1407840	1425900	25541	2%	10%
1405040	1425620	29105	2%	-6%
1290520	1309420	26729	2%	5%
1305920	1274000	45142	4%	7%
400400	400820	594	0%	0%
1484000	1493240	13067	1%	7%
4416720	4504780	124536	3%	11%
406560	387800	26531	7%	-3%
366800	329280	53061	16%	4%
87080	84700	3366	4%	25%
1586200	1567020	27125	2%	-4%
4157160	4128880	39994	1%	-1%
898240	851340	66327	8%	-2%
1540000	1502900	52467	3%	15%
1473920	1471260	3762	0%	-3%
500640	502740	2970	1%	1%
1184680	1136660	67911	6%	0%
1829520	1739780	126912	7%	-5%
629720	622860	9702	2%	3%
3413760	3233300	255209	8%	0%
983080	985880	3960	0%	4%
2227120	2119320	152452	7%	5%
1426880	1415540	16037	1%	1%
382760	387240	6336	2%	8%

362320	346780	21977	6%	6%
3019240	2997120	31282	1%	18%
5928720	5768420	226698	4%	0%
2896040	2930900	49299	2%	-2%
623840	597800	36826	6%	10%
564480	564340	198	0%	27%
4013240	3985380	39400	1%	5%
1348480	1353660	7326	1%	-4%
770000	756420	19205	3%	15%
151200	148400	3960	3%	8%
1723680	1677480	65337	4%	-6%
1001000	999320	2376	0%	2%
1225840	1220940	6930	1%	5%
263200	254520	12275	5%	10%
1007720	1014860	10097	1%	-2%
45640	43400	3168	7%	8%
2810080	2826600	23363	1%	4%
1662640	1606500	79394	5%	3%
191800	187600	5940	3%	6%
494200	484680	13463	3%	13%
569520	582120	17819	3%	10%
40320	41440	1584	4%	-3%
140840	139720	1584	1%	6%
559160	539280	28115	5%	23%
663600	658840	6732	1%	-1%
4554760	4468800	121566	3%	4%
539560	485100	77018	16%	-1%
1766800	1728300	54447	3%	10%
537600	547400	13859	3%	19%
1245720	1292060	65535	5%	23%
652400	682780	42964	6%	14%
1989680	1886780	145523	8%	8%
264600	274400	13859	5%	1%
1429120	1469300	56823	4%	0%
2506000	2554580	68702	3%	13%
358960	363720	6732	2%	9%
3622080	3547320	105727	3%	7%
1116080	1100820	21581	2%	7%
458640	449680	12671	3%	26%
4224080	4186000	53853	1%	10%
652120	643020	12869	2%	5%
3043040	3051720	12275	0%	5%
1346240	1310960	49893	4%	20%
73080	68460	6534	10%	6%
710640	712740	2970	0%	-5%
807520	765380	59595	8%	1%
1383760	1408260	34648	2%	3%
135240	134960	396	0%	23%
1758400	1773800	21779	1%	8%
3348800	3351180	3366	0%	1%
461720	452480	13067	3%	18%
245000	252280	10295	4%	6%

869400	850780	26333	3%	3%
621880	583100	54843	9%	17%
679280	709100	42172	6%	2%
2730280	2625420	148294	6%	5%
3049480	3067260	25145	1%	2%
947240	972720	36034	4%	3%
322280	329700	10493	3%	15%
143640	142100	2178	2%	10%
1341480	1327200	20195	2%	6%
3967040	4045440	110874	3%	17%
3531080	3525200	8316	0%	12%
71680	71400	396	1%	7%
1081360	1027320	76424	7%	14%
3164560	3195780	44152	1%	7%
2859920	2883860	33856	1%	5%
3926440	4039980	160570	4%	-1%
204400	212240	11087	5%	-1%
230440	225680	6732	3%	-2%
1431640	1443960	17423	1%	11%
32200	32200	0	0%	-2%
38080	41160	4356	11%	6%
212240	221200	12671	6%	-1%
284200	287980	5346	2%	-3%
256480	261100	6534	3%	3%
562520	560840	2376	0%	0%
1697360	1640800	79988	5%	-3%
5039720	5051060	16037	0%	1%
1223320	1249500	37024	3%	4%
271880	270200	2376	1%	-1%
1673280	1754200	114438	7%	-2%
863240	881720	26135	3%	6%
131040	131600	792	1%	1%
1335880	1363180	38608	3%	3%
186480	187040	792	0%	-4%
346080	348740	3762	1%	2%
897960	876400	30490	3%	3%
2964360	2949520	20987	1%	7%
2683240	2660280	32470	1%	2%
969920	981540	16433	2%	1%
227920	221060	9702	4%	5%
395360	396060	990	0%	22%
1481200	1479520	2376	0%	-1%
297640	296100	2178	1%	-8%
511280	504840	9108	2%	11%
675360	686420	15641	2%	0%
400960	410620	13661	3%	2%
982520	921900	85730	9%	10%
1001560	995120	9108	1%	-9%
1223320	1246140	32272	3%	16%
916720	943180	37420	4%	5%
551880	584080	45538	8%	-2%
290080	292320	3168	1%	-11%

889560	871780	25145	3%	1%
377720	370300	10493	3%	-7%
3714480	3719520	7128	0%	15%
41440	42840	1980	5%	13%
1592920	1620220	38608	2%	0%
1122800	1131620	12473	1%	0%
190400	182840	10691	6%	2%
580160	590240	14255	2%	32%
2179800	2115680	90679	4%	2%
1976240	2115680	197198	9%	1%
181440	183120	2376	1%	15%
2395400	2417240	30886	1%	1%
58240	56280	2772	5%	5%
433440	415800	24947	6%	5%
484120	476420	10889	2%	1%
1196440	1171240	35638	3%	10%
4833080	4830420	3762	0%	8%
1352120	1355900	5346	0%	21%
1701560	1592640	154036	10%	-1%
822080	816340	8118	1%	7%
162680	175140	17621	10%	1%
49280	47040	3168	7%	6%
442400	426860	21977	5%	19%
4262440	4134900	180369	4%	6%
3554320	3527720	37618	1%	2%

Compound Name	Assay Name	Assay Format	Assay Target	Result Type	RC50 (uM)	Hill	Curve Bottom	Curve Top	Max Response	Result Graph
PACAP-27	Arrestin	Agonist	ADCYAP1R1	EC50	0.0013353	1.41	-2.2	100	101.6	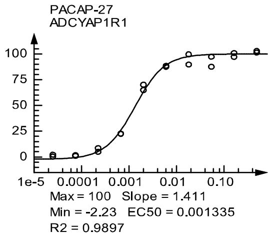
2-Cl-IB-MECA	Arrestin	Agonist	ADORA3	EC50	0.01014	1.27	-0.8	98.7	98.642	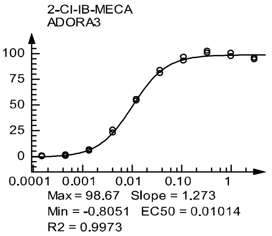
Phenylephrine	Arrestin	Agonist	ADRA1B	EC50	0.01996	0.89	-0.3	100	100.39	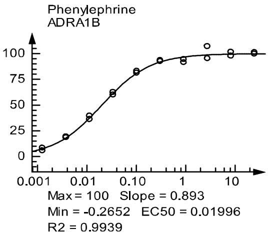
UK 14,304	Arrestin	Agonist	ADRA2A	EC50	0.0034478	0.67	0	102.3	100.15	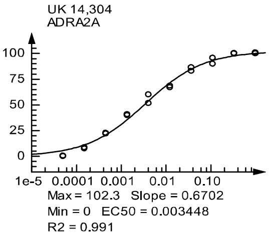
UK 14,304	Arrestin	Agonist	ADRA2B	EC50	0.15441	1.23	-1.9	105	101.03	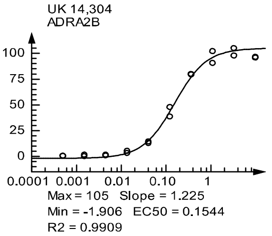
UK 14,304	Arrestin	Agonist	ADRA2C	EC50	0.086042	1.11	-1.6	100	100.15	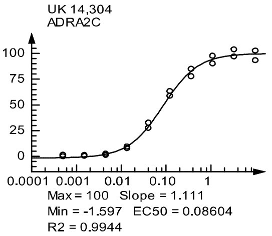
Isoproterenol	Arrestin	Agonist	ADRB1	EC50	0.031895	1.47	0	95.1	98.333	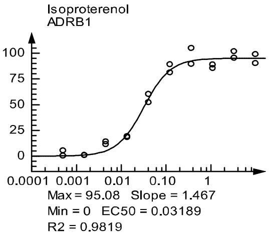

Compound Name	Assay Name	Assay Format	Assay Target	Result Type	RC50 (uM)	Hill	Curve Bottom	Curve Top	Max Response	Result Graph
Isoproterenol	Arrestin	Agonist	ADRB2	EC50	0.039342	1.21	0	98.7	98.541	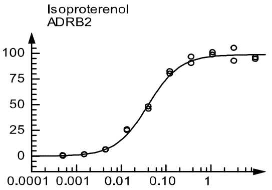 Max = 98.69 Slope = 1.212<br>Min = 0.01921 EC50 = 0.03934<br>R2 = 0.9936
Angiotensin II	Arrestin	Agonist	AGTR1	EC50	0.00034554	1.45	0.8	96.7	101.38	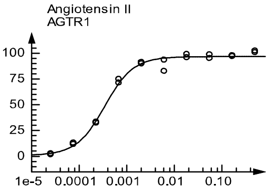 Max = 96.68 Slope = 1.454<br>Min = 0.7937 EC50 = 0.0003455<br>R2 = 0.9901
Apelin-13	Arrestin	Agonist	AGTRL1	EC50	0.00098737	1.46	1.4	103.3	106.18	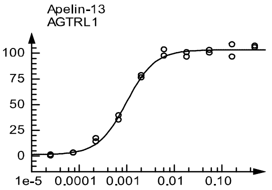 Max = 103.3 Slope = 1.46<br>Min = 1.381 EC50 = 0.0009874<br>R2 = 0.9937
Vasopressin	Arrestin	Agonist	AVPR1A	EC50	0.0011365	0.83	0	106	115.68	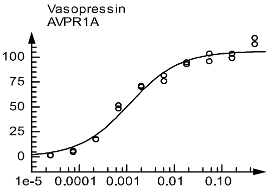 Max = 106 Slope = 0.8294<br>Min = 0 EC50 = 0.001137<br>R2 = 0.9775
Vasopressin	Arrestin	Agonist	AVPR1B	EC50	0.001104	0.8	0	99	101.25	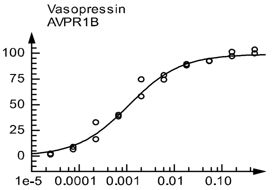 Max = 99.03 Slope = 0.8029<br>Min = 0 EC50 = 0.001104<br>R2 = 0.9831
Vasopressin	Arrestin	Agonist	AVPR2	EC50	0.00045952	1.58	2.5	98.5	105.18	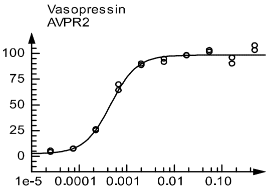 Max = 98.46 Slope = 1.577<br>Min = 2.506 EC50 = 0.0004595<br>R2 = 0.9903
LDA-Bradykinin	Arrestin	Agonist	BDKRB1	EC50	0.0016991	1.16	10.2	100	121.5	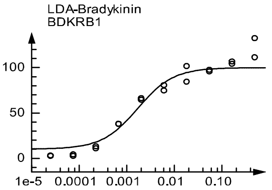 Max = 100 Slope = 1.159<br>Min = 10.17 EC50 = 0.001699<br>R2 = 0.9452

Compound Name	Assay Name	Assay Format	Assay Target	Result Type	RC50 (uM)	Hill	Curve Bottom	Curve Top	Max Response	Result Graph
Bradykinin	Arrestin	Agonist	BDKRB2	EC50	0.00075915	1.59	3.1	100	101.16	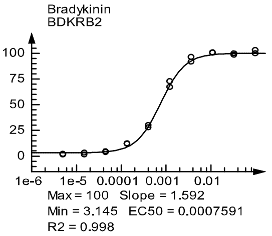
TAPN-Bombesin	Arrestin	Agonist	BRS3	EC50	0.0017867	0.66	0	94.6	100	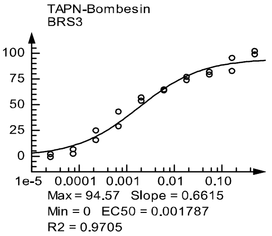
C3A Receptor Agonist (Short Fragment)	Arrestin	Agonist	C3AR1	EC50	0.089023	1.21	0	95.6	98.996	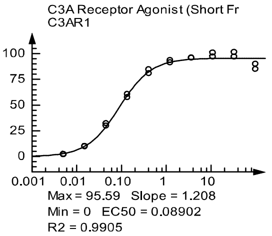
Complement C5a	Arrestin	Agonist	C5AR1	EC50	0.00062959	1.87	0.1	100.9	102.7	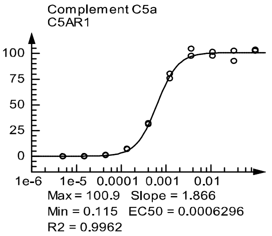
Complement C5a	Arrestin	Agonist	C5L2	EC50	0.0012399	1.97	0	102.2	100.18	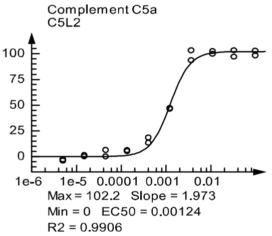
Calcitonin	Arrestin	Agonist	CALCR	EC50	0.030968	1.32	0.6	100	103.36	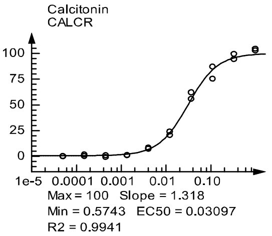
beta CGRP	Arrestin	Agonist	CALCRL-RAMP1	EC50	0.00043504	1.69	0	91.2	91.591	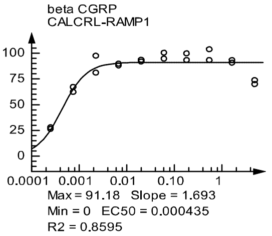

Compound Name	Assay Name	Assay Format	Assay Target	Result Type	RC50 (uM)	Hill	Curve Bottom	Curve Top	Max Response	Result Graph
Adrenomedullin	Arrestin	Agonist	CALCRL-RAMP2	EC50	0.043792	1.14	2.6	105	102.35	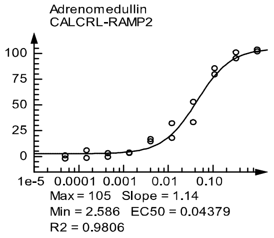
Adrenomedullin	Arrestin	Agonist	CALCRL-RAMP3	EC50	0.0013667	1.19	-2.9	100	100.3	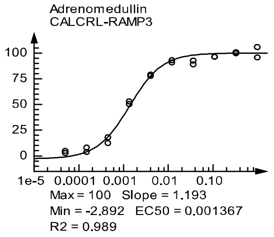
Calcitonin	Arrestin	Agonist	CALCR-RAMP2	EC50	0.007865	0.81	-3.1	100	102.13	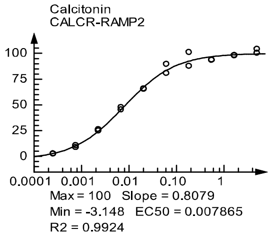
Calcitonin	Arrestin	Agonist	CALCR-RAMP3	EC50	0.10893	1.55	12.3	100	132.46	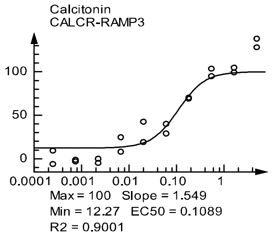
CCK-8	Arrestin	Agonist	CCKAR	EC50	0.0026133	1.25	-2.5	100	100.39	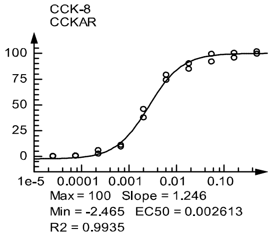
CCK-8	Arrestin	Agonist	CCKBR	EC50	0.00032724	2.33	4.2	97.6	98.585	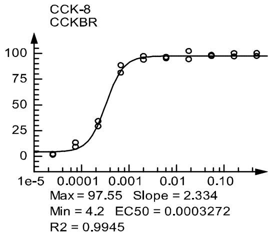
CCL27	Arrestin	Agonist	CCR10	EC50	0.014494	1.19	-1.7	101.1	105.15	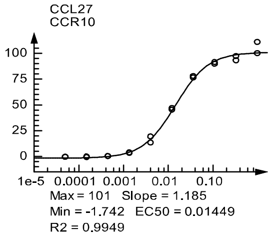

Compound Name	Assay Name	Assay Format	Assay Target	Result Type	RC50 (uM)	Hill	Curve Bottom	Curve Top	Max Response	Result Graph
CCL3	Arrestin	Agonist	CCR1	EC50	0.0022788	1.19	0	94	104.57	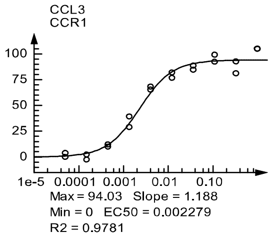
CCL2	Arrestin	Agonist	CCR2	EC50	0.0046831	0.9	-0.6	103.7	100.52	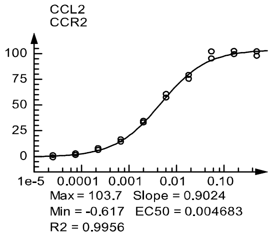
CCL13	Arrestin	Agonist	CCR3	EC50	0.022282	1.08	-4	107.5	104.53	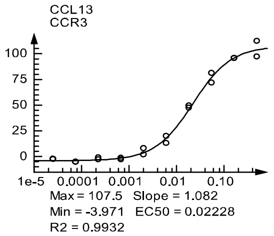
CCL22	Arrestin	Agonist	CCR4	EC50	0.0013346	0.7	-0.2	100	101.93	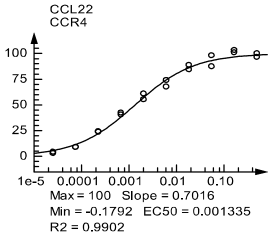
CCL3	Arrestin	Agonist	CCR5	EC50	0.01626	1.09	0.2	105.3	102.15	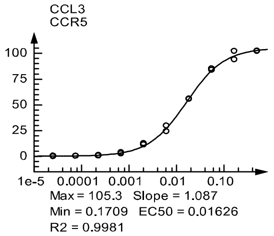
CCL20	Arrestin	Agonist	CCR6	EC50	0.0090203	0.79	-2.5	106.2	100.15	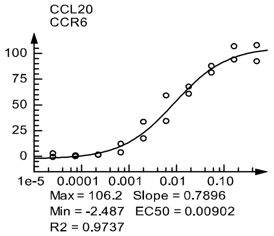
CCL19	Arrestin	Agonist	CCR7	EC50	0.0090459	1.65	1.4	101.1	104.39	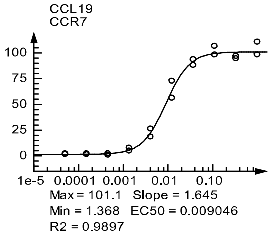

Compound Name	Assay Name	Assay Format	Assay Target	Result Type	RC50 (uM)	Hill	Curve Bottom	Curve Top	Max Response	Result Graph
CCL1	Arrestin	Agonist	CCR8	EC50	0.055652	1.28	0.2	106.1	102.8	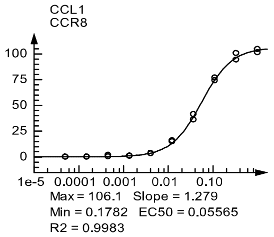
CCL25	Arrestin	Agonist	CCR9	EC50	0.14796	1.29	1.6	100	100	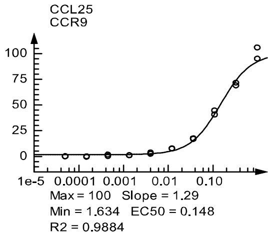
Acetylcholine	Arrestin	Agonist	CHRM1	EC50	1.1988	0.62	-6	100	101.24	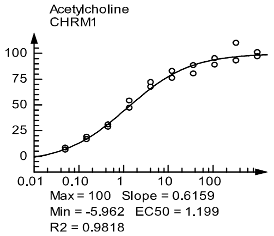
Acetylcholine	Arrestin	Agonist	CHRM2	EC50	2.4133	1	-5.2	100	103.07	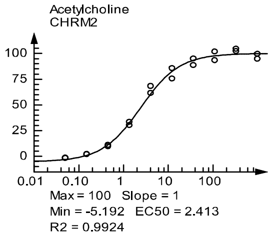
Acetylcholine	Arrestin	Agonist	CHRM3	EC50	0.36277	0.67	0	102.2	102.33	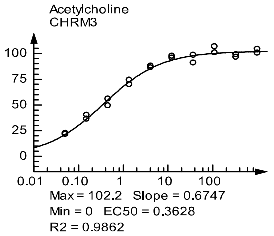
Acetylcholine	Arrestin	Agonist	CHRM4	EC50	0.85906	1.18	-2.1	102	100.62	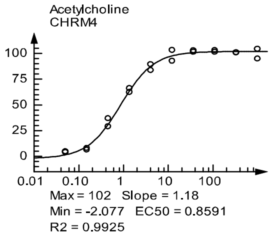
Acetylcholine	Arrestin	Agonist	CHRM5	EC50	2.1473	1.02	0	96.3	94.091	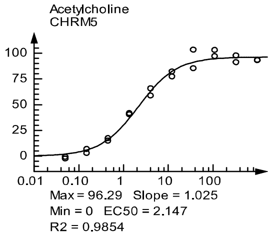

Compound Name	Assay Name	Assay Format	Assay Target	Result Type	RC50 (uM)	Hill	Curve Bottom	Curve Top	Max Response	Result Graph
Chemerin	Arrestin	Agonist	CMKLR1	EC50	0.0010713	1.52	-0.8	98	103.16	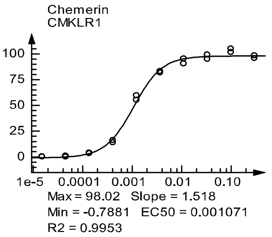
CP55940	Arrestin	Agonist	CNR1	EC50	0.0026278	0.84	0	97	100	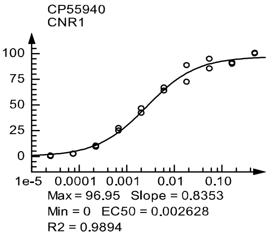
CP55940	Arrestin	Agonist	CNR2	EC50	0.0025322	2.25	0	108.4	120.68	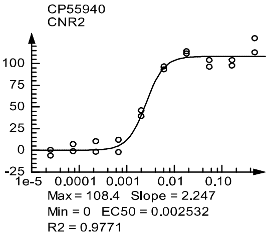
Sauvagine	Arrestin	Agonist	CRHR1	EC50	0.0013292	1.77	0	96.6	99.189	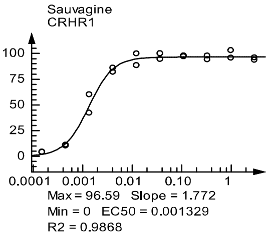
Sauvagine	Arrestin	Agonist	CRHR2	EC50	0.0037467	1.51	-1.1	96.7	96.544	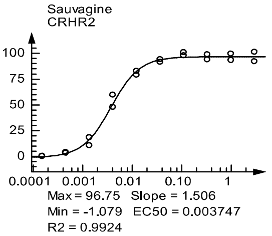
PGD2	Arrestin	Agonist	CRTH2	EC50	0.011936	0.96	0	100.3	99.225	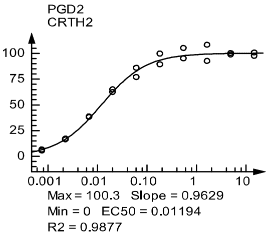
Fractalkine	Arrestin	Agonist	CX3CR1	EC50	0.00030093	1.64	0.5	99.4	101.54	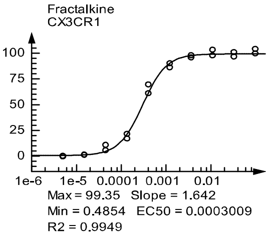

Compound Name	Assay Name	Assay Format	Assay Target	Result Type	RC50 (uM)	Hill	Curve Bottom	Curve Top	Max Response	Result Graph
CXCL8	Arrestin	Agonist	CXCR1	EC50	0.0033408	1.22	-0.7	99.8	100.9	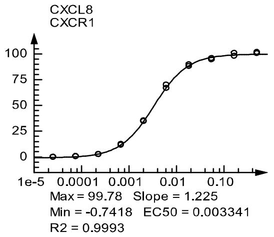
CXCL8	Arrestin	Agonist	CXCR2	EC50	0.00058784	0.9	-4.1	98.8	101.28	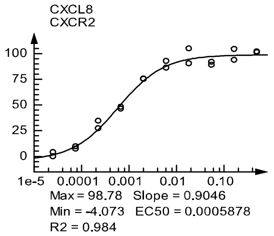
CXCL11	Arrestin	Agonist	CXCR3	EC50	0.016801	1.05	-2.2	105.3	104.79	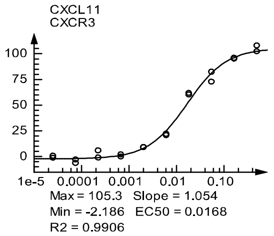
CXCL12	Arrestin	Agonist	CXCR4	EC50	0.0023134	1.58	9.5	103.6	109.74	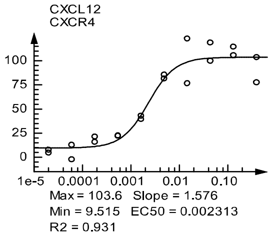
CXCL13	Arrestin	Agonist	CXCR5	EC50	0.055638	1.3	2.3	100	100	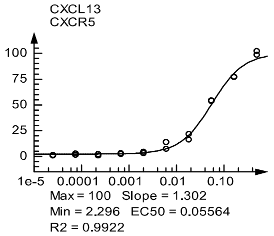
CXCL16	Arrestin	Agonist	CXCR6	EC50	0.0008179	1.34	1.2	109.1	139.6	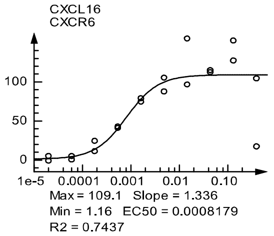
CXCL12	Arrestin	Agonist	CXCR7	EC50	0.01211	1.45	-1.2	102.2	100.98	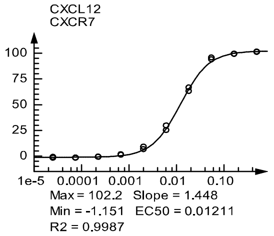

Compound Name	Assay Name	Assay Format	Assay Target	Result Type	RC50 (uM)	Hill	Curve Bottom	Curve Top	Max Response	Result Graph
Dopamine	Arrestin	Agonist	DRD1	EC50	0.25865	1.13	-1.2	100	100	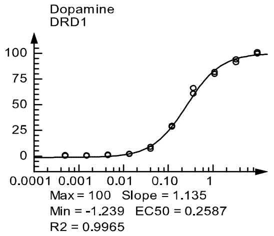
Dopamine	Arrestin	Agonist	DRD2L	EC50	0.04272	1.2	0	102.1	101.55	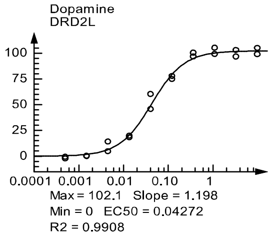
Dopamine	Arrestin	Agonist	DRD2S	EC50	0.069242	1.4	0	101.8	102.85	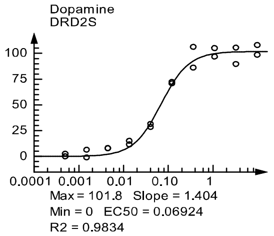
Dopamine	Arrestin	Agonist	DRD3	EC50	0.0047447	1.37	0	94.8	102.2	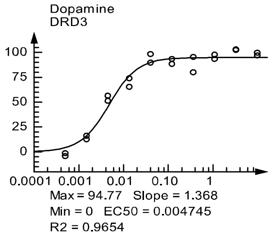
Dopamine	Arrestin	Agonist	DRD4	EC50	0.045174	1.38	0	99.1	101.41	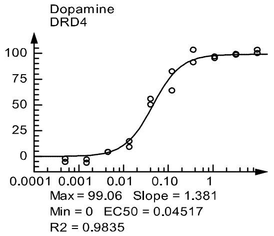
Dopamine	Arrestin	Agonist	DRD5	EC50	0.089551	1.14	-0.9	101.1	101.24	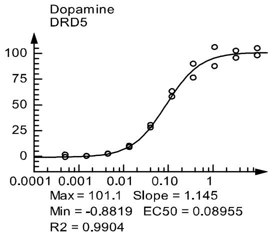
7a,25-Dihydroxycholesterol	Arrestin	Agonist	EBI2	EC50	0.36238	0.55	5	104.1	102.44	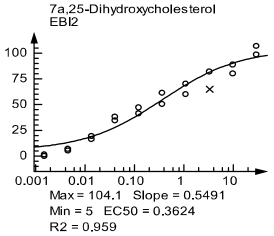

Compound Name	Assay Name	Assay Format	Assay Target	Result Type	RC50 (uM)	Hill	Curve Bottom	Curve Top	Max Response	Result Graph
S-1-P	Arrestin	Agonist	EDG1	EC50	0.010881	0.67	0	95.2	100.87	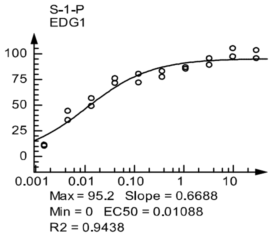
S-1-P	Arrestin	Agonist	EDG3	EC50	0.0045497	1.23	0	99.2	102.23	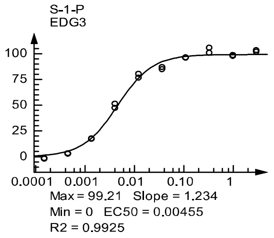
Oleoyl LPA	Arrestin	Agonist	EDG4	EC50	0.64	1.04	-0.4	100	114.24	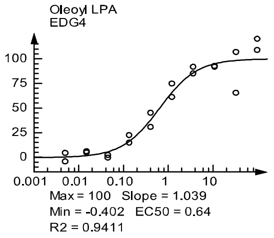
S-1-P	Arrestin	Agonist	EDG5	EC50	0.028036	2.23	0	104.3	100.65	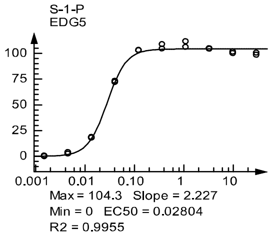
S-1-P	Arrestin	Agonist	EDG6	EC50	0.11169	0.63	11.6	110	132.13	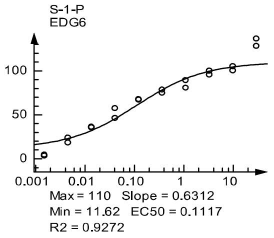
Oleoyl LPA	Arrestin	Agonist	EDG7	EC50	0.27782	0.91	-7.2	105	102.41	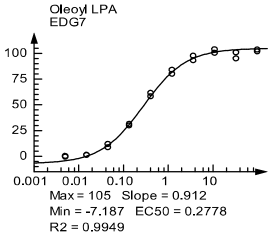
Endothelin I	Arrestin	Agonist	EDNRA	EC50	0.0031472	1.23	-4.4	100	101.77	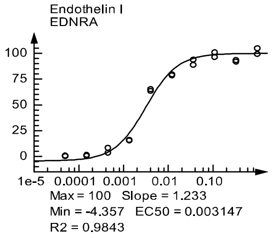

Compound Name	Assay Name	Assay Format	Assay Target	Result Type	RC50 (uM)	Hill	Curve Bottom	Curve Top	Max Response	Result Graph
Endothelin 3	Arrestin	Agonist	EDNRB	EC50	0.0057293	1.56	-1	99.3	103.26	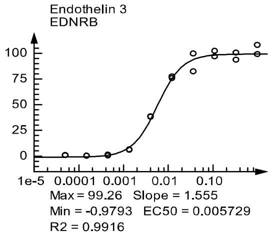
TFLLR-NH2	Arrestin	Agonist	F2R	EC50	11.119	0.75	0	117.2	102	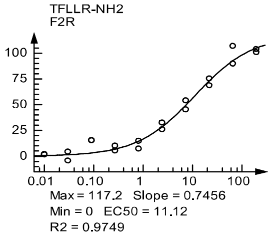
SLIGRL-NH2	Arrestin	Agonist	F2RL1	EC50	1.1573	0.94	-1.6	105	101.03	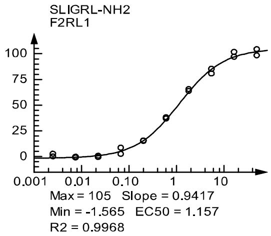
AYPGKF-NH2	Arrestin	Agonist	F2RL3	EC50	3.6838	1.92	0	96.6	104.84	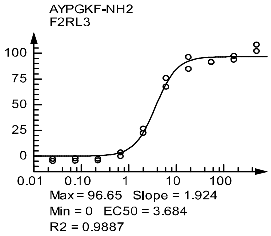
GW9508	Arrestin	Agonist	FFAR1	EC50	0.85232	0.92	-8	100	105.29	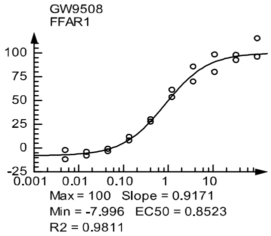
WKYMVm-NH2	Arrestin	Agonist	FPR1	EC50	0.0018579	0.88	-9.2	105	103.56	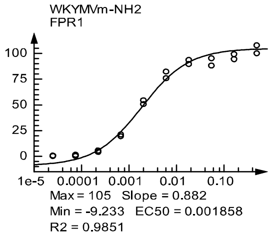
WKYMVm-NH2	Arrestin	Agonist	FPRL1	EC50	0.00077485	1.39	-5.3	104	103.15	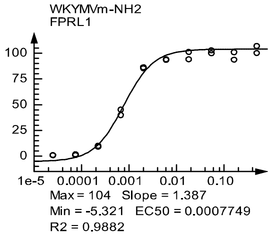

Compound Name	Assay Name	Assay Format	Assay Target	Result Type	RC50 (uM)	Hill	Curve Bottom	Curve Top	Max Response	Result Graph
FSH	Arrestin	Agonist	FSHR	EC50	0.0023329	1.16	-1.1	102.9	100.62	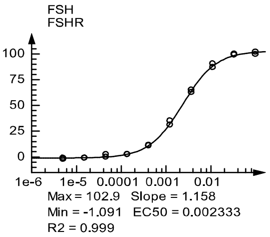
Galanin	Arrestin	Agonist	GALR1	EC50	0.00087378	1.43	-6.4	100	99.766	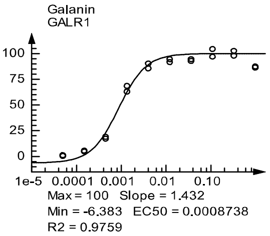
Galanin	Arrestin	Agonist	GALR2	EC50	0.0065161	0.96	0	95.9	102.61	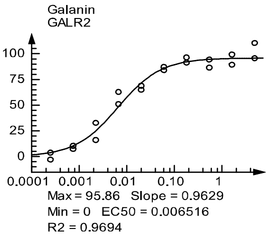
Glucagon	Arrestin	Agonist	GCGR	EC50	0.001929	2.15	0	97.2	98.909	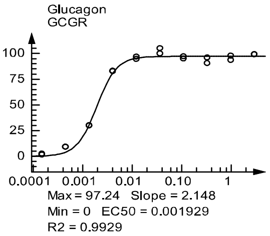
Ghrelin	Arrestin	Agonist	GHSR	EC50	0.003451	1.98	-1.5	100	103.5	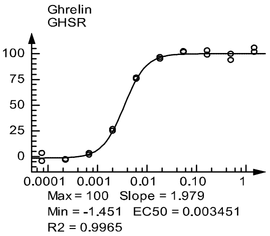
GIP	Arrestin	Agonist	GIPR	EC50	0.0050659	1.13	0	103.5	100.4	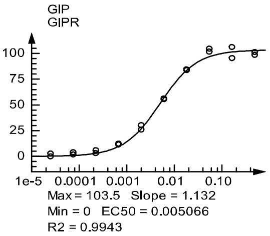
Exendin-4	Arrestin	Agonist	GLP1R	EC50	0.0031748	1.25	0	97.8	98.14	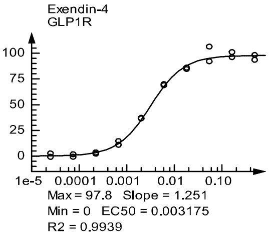

Compound Name	Assay Name	Assay Format	Assay Target	Result Type	RC50 (uM)	Hill	Curve Bottom	Curve Top	Max Response	Result Graph
GLP II (1-33)	Arrestin	Agonist	GLP2R	EC50	0.0017703	0.81	0	101.8	100.57	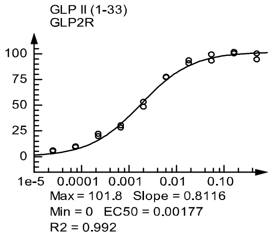
Chemerin	Arrestin	Agonist	GPR1	EC50	0.0027699	1.82	-0.3	100	101.79	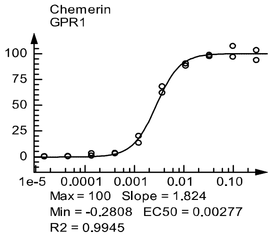
QRFP-26	Arrestin	Agonist	GPR103	EC50	0.0060542	1.16	-2.2	100	101.04	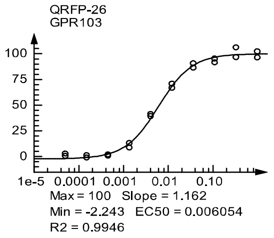
Nicotinic Acid	Arrestin	Agonist	GPR109A	EC50	3.434	1.17	-9.2	100	100	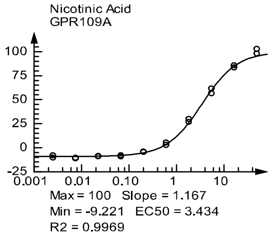
3-Hydroxyoctanoic Acid	Arrestin	Agonist	GPR109B	EC50	280.23	1.23	0.2	115	110.57	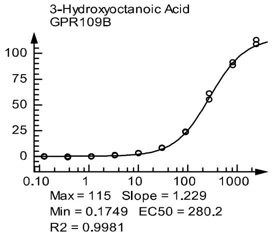
Oleoyl Ethanolamide	Arrestin	Agonist	GPR119	EC50	2.1337	1.12	1	94.1	99.664	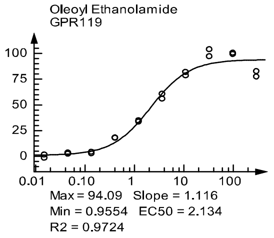
GW9508	Arrestin	Agonist	GPR120	EC50	5.6114	1.14	-2.8	100	100	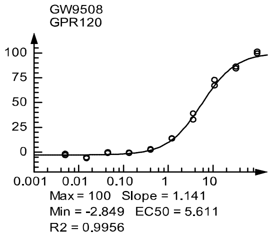

Compound Name	Assay Name	Assay Format	Assay Target	Result Type	RC50 (uM)	Hill	Curve Bottom	Curve Top	Max Response	Result Graph
Zaprinast	Arrestin	Agonist	GPR35	EC50	2.0833	0.76	-1.5	110	109.37	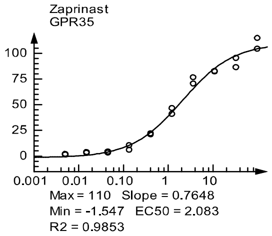
Oleoyl LPA	Arrestin	Agonist	GPR92	EC50	0.51832	1.23	4.1	102	109.29	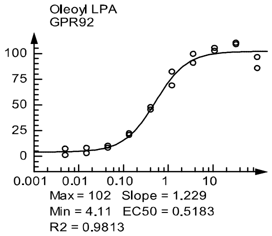
GRP	Arrestin	Agonist	GRPR	EC50	0.00076819	1.93	0	99.4	102.91	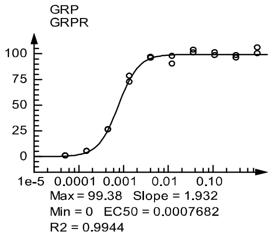
Orexin A	Arrestin	Agonist	HCRTR1	EC50	0.005905	1.66	-0.7	96.3	100.4	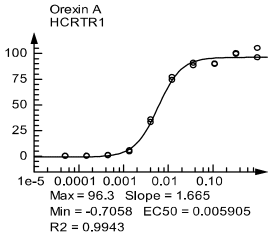
Orexin A	Arrestin	Agonist	HCRTR2	EC50	0.011861	1.37	-0.2	101.6	102.36	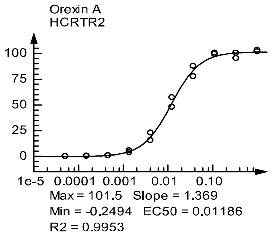
Histamine	Arrestin	Agonist	HRH1	EC50	0.080052	0.85	0	98.7	103.06	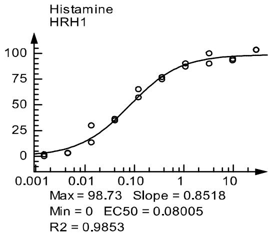
Histamine	Arrestin	Agonist	HRH2	EC50	5.4194	0.98	0.6	110	106.03	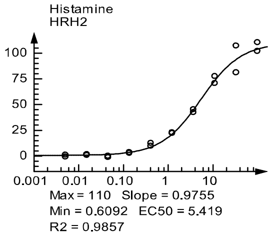

Compound Name	Assay Name	Assay Format	Assay Target	Result Type	RC50 (uM)	Hill	Curve Bottom	Curve Top	Max Response	Result Graph
R-a methylhistamine	Arrestin	Agonist	HRH3	EC50	0.59925	1.24	1.3	105	105.32	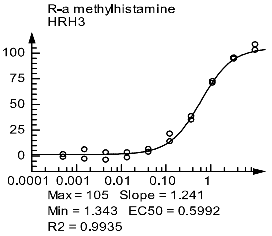
Histamine	Arrestin	Agonist	HRH4	EC50	0.055254	1.13	4.2	103.1	101.53	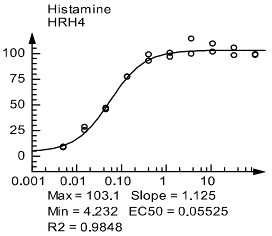
Serotonin / 5-HT	Arrestin	Agonist	HTR1A	EC50	0.019443	1.38	-3.1	103	101.82	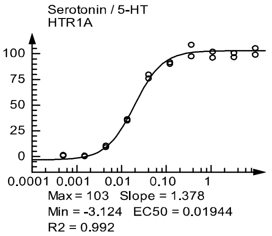
Serotonin / 5-HT	Arrestin	Agonist	HTR1B	EC50	0.053264	1.19	3.8	100.8	101.35	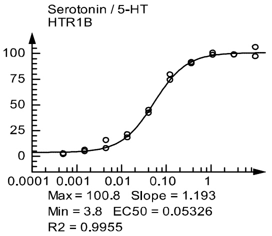
Serotonin / 5-HT	Arrestin	Agonist	HTR1E	EC50	0.011451	1.07	0	95.1	102.22	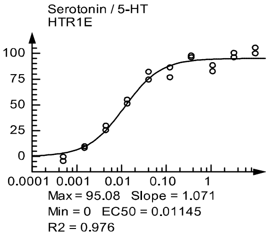
Serotonin / 5-HT	Arrestin	Agonist	HTR1F	EC50	0.041658	1.2	-0.4	99.7	102.62	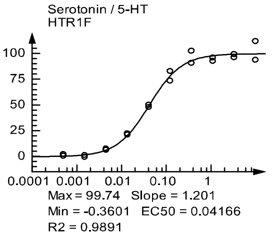
Serotonin / 5-HT	Arrestin	Agonist	HTR2A	EC50	0.03358	0.92	4.4	100	101.85	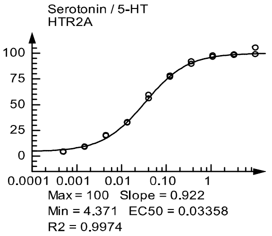

Compound Name	Assay Name	Assay Format	Assay Target	Result Type	RC50 (uM)	Hill	Curve Bottom	Curve Top	Max Response	Result Graph
Serotonin / 5-HT	Arrestin	Agonist	HTR2C	EC50	0.0024021	1.1	0	99.2	103.81	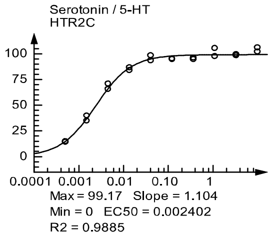
Serotonin / 5-HT	Arrestin	Agonist	HTR5A	EC50	0.0013757	1.22	0	92.2	84.655	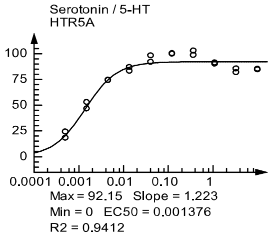
Kisspeptin-10	Arrestin	Agonist	KISS1R	EC50	0.021634	1.15	-0.1	99.8	101.86	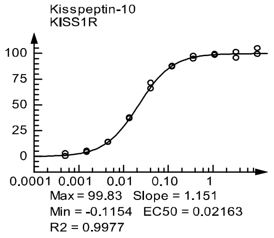
hCG	Arrestin	Agonist	LHCGR	EC50	0.0024713	0.61	0	104.4	99.999	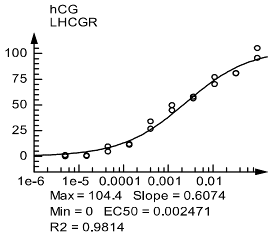
Leukotriene B4	Arrestin	Agonist	LTB4R	EC50	0.2565	0.52	0	119.7	106.37	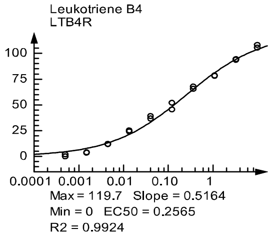
Melanotan II	Arrestin	Agonist	MC1R	EC50	0.00019251	1.06	0	96.8	102.75	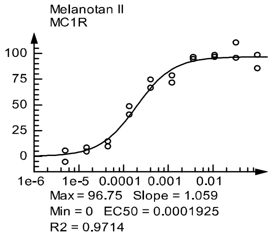
Melanotan II	Arrestin	Agonist	MC3R	EC50	0.0022356	0.8	-0.6	100	99.998	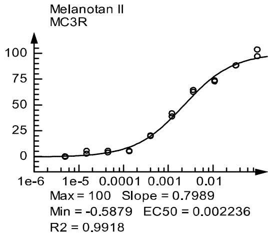

Compound Name	Assay Name	Assay Format	Assay Target	Result Type	RC50 (uM)	Hill	Curve Bottom	Curve Top	Max Response	Result Graph
Melanotan II	Arrestin	Agonist	MC4R	EC50	0.0011304	1.05	0.2	101.7	100.96	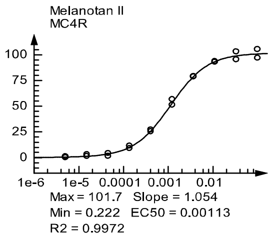
Melanotan II	Arrestin	Agonist	MC5R	EC50	0.0057816	0.74	0	101.8	103.84	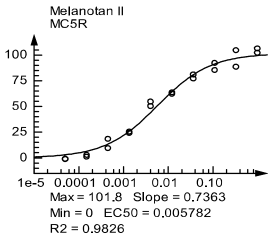
MCH	Arrestin	Agonist	MCHR1	EC50	0.092666	1.15	-1.5	105	100	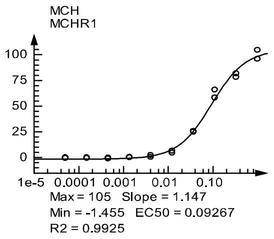
MCH	Arrestin	Agonist	MCHR2	EC50	0.0069126	1.21	-0.4	100	99.612	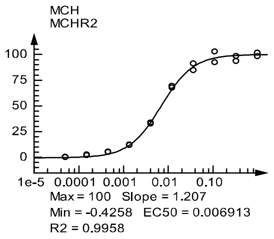
Motilin	Arrestin	Agonist	MLNR	EC50	0.00041096	1.22	0	97.5	98.476	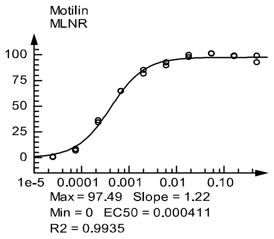
BAM(8-22)	Arrestin	Agonist	MRGPRX1	EC50	3.4396	0.78	0	115.7	100	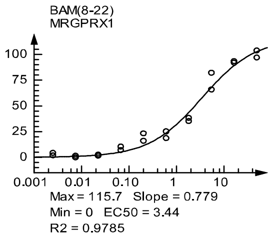
Cortistatin 14	Arrestin	Agonist	MRGPRX2	EC50	0.4622	0.86	0	103.8	100	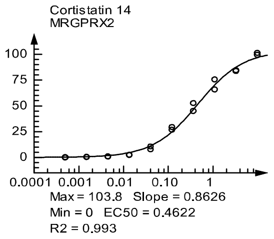

Compound Name	Assay Name	Assay Format	Assay Target	Result Type	RC50 (uM)	Hill	Curve Bottom	Curve Top	Max Response	Result Graph
2-Iodomelatonin	Arrestin	Agonist	MTNR1A	EC50	0.00047149	1.33	0	95.4	102.5	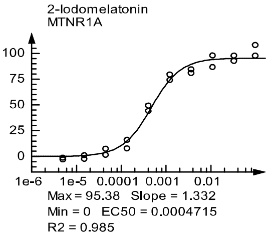
Neuromedin B	Arrestin	Agonist	NMBR	EC50	0.0057376	1.31	0	95.9	102.51	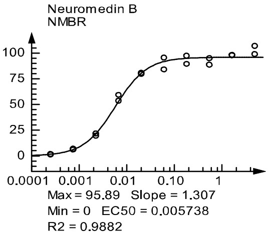
Neuromedin U-25	Arrestin	Agonist	NMU1R	EC50	0.001618	1.37	0	94	99.59	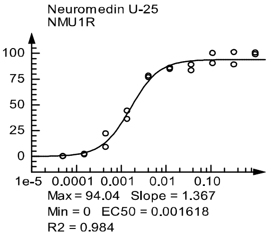
Neuropeptide W23	Arrestin	Agonist	NPBWR1	EC50	0.0015613	1.08	0	92.4	100.95	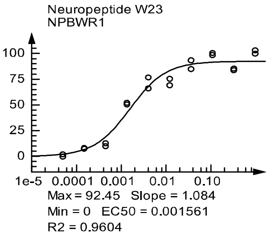
Neuropeptide W23	Arrestin	Agonist	NPBWR2	EC50	0.0021084	2.61	0.2	97.2	97.813	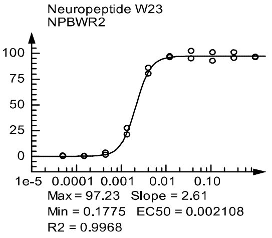
RFRP-3	Arrestin	Agonist	NPFFR1	EC50	0.07765	0.85	0	102.2	102.59	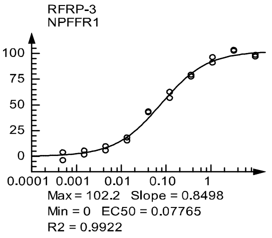
Neuropeptide S	Arrestin	Agonist	NPSR1b	EC50	0.012328	1.07	-5	94.5	99.255	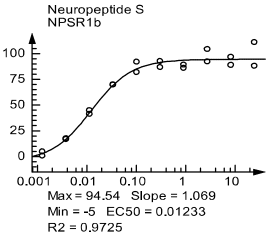

Compound Name	Assay Name	Assay Format	Assay Target	Result Type	RC50 (uM)	Hill	Curve Bottom	Curve Top	Max Response	Result Graph
Peptide YY	Arrestin	Agonist	NPY1R	EC50	0.0043249	1.09	0	97.9	101.6	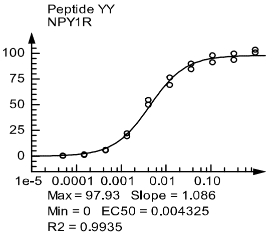
Peptide YY	Arrestin	Agonist	NPY2R	EC50	0.0032503	2.08	0.1	97.7	99.012	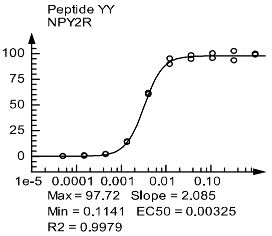
[Lys 8,9] Neurotensin	Arrestin	Agonist	NTSR1	EC50	0.00011679	1.53	0	98.1	97.401	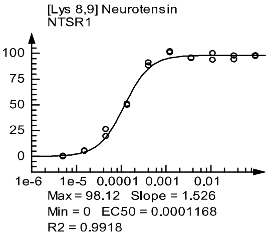
DADLE	Arrestin	Agonist	OPRD1	EC50	0.0015313	1.43	0	97.9	100.83	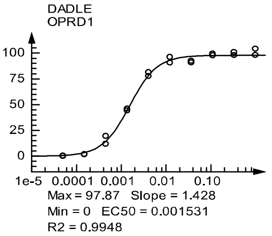
Dynorphin A	Arrestin	Agonist	OPRK1	EC50	0.020336	1.29	-2.9	100	97.271	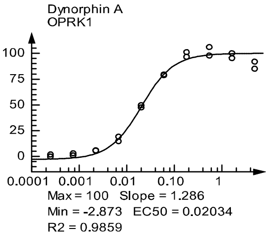
Orphanin FQ	Arrestin	Agonist	OPRL1	EC50	0.0092907	1.08	0	99.3	98.702	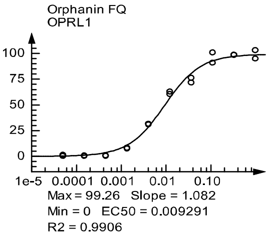
[Met] Enkephalin	Arrestin	Agonist	OPRM1	EC50	0.59919	0.78	-7	105	104.46	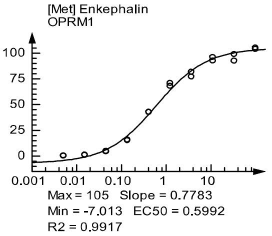

Compound Name	Assay Name	Assay Format	Assay Target	Result Type	RC50 (uM)	Hill	Curve Bottom	Curve Top	Max Response	Result Graph
5-OxoETE	Arrestin	Agonist	OXER1	EC50	1.7094	0.78	2.6	110	99.998	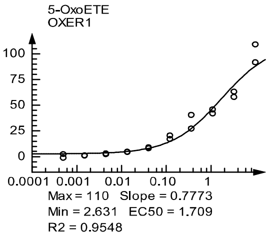
Oxytocin	Arrestin	Agonist	OXTR	EC50	0.0029786	0.92	-0.5	105	100	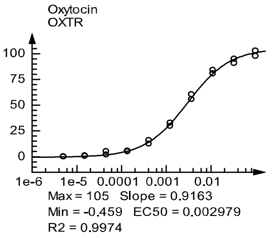
2-methylthio-ADP	Arrestin	Agonist	P2RY1	EC50	0.01331	0.91	0	96.5	102.53	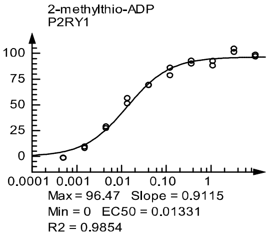
ATP	Arrestin	Agonist	P2RY11	EC50	388.45	2.75	3.4	103	100	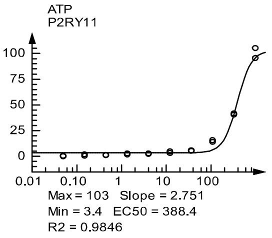
2-methylthio-ADP	Arrestin	Agonist	P2RY12	EC50	0.00091186	0.97	0	94.2	93.517	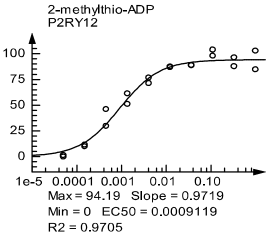
UTP	Arrestin	Agonist	P2RY2	EC50	2.1196	1.31	-2.3	105	102.62	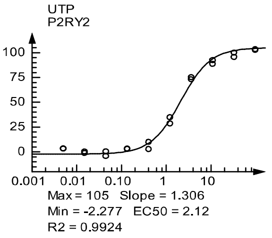
UTP	Arrestin	Agonist	P2RY4	EC50	0.30508	0.98	0	100.6	100.87	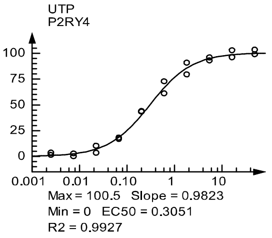

Compound Name	Assay Name	Assay Format	Assay Target	Result Type	RC50 (uM)	Hill	Curve Bottom	Curve Top	Max Response	Result Graph
UDP	Arrestin	Agonist	P2RY6	EC50	0.023688	1.24	-0.2	98.5	100.23	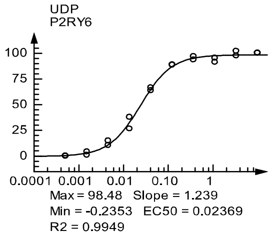
Pancreatic Polypeptide	Arrestin	Agonist	PPYR1	EC50	0.0016233	1.57	-0.4	98.1	98.553	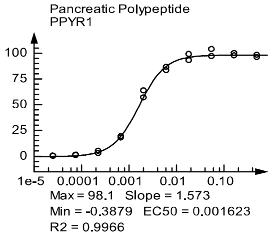
PrRP-31	Arrestin	Agonist	PRLHR	EC50	0.0017097	1.44	0	96.9	98.988	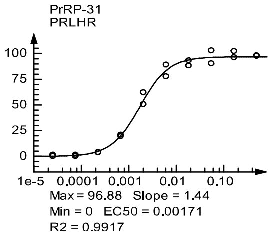
EG VEGF	Arrestin	Agonist	PROKR1	EC50	0.025431	1.06	-0.2	110	107.75	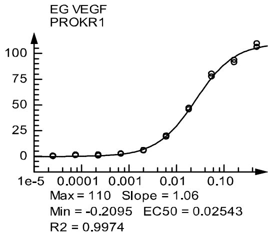
EG VEGF	Arrestin	Agonist	PROKR2	EC50	0.015632	1.45	1.3	100	107.2	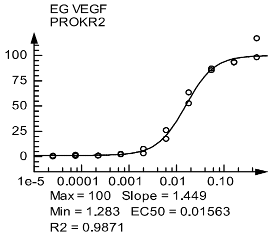
PAF	Arrestin	Agonist	PTAFR	EC50	0.005423	1.56	-2.2	99.1	101.49	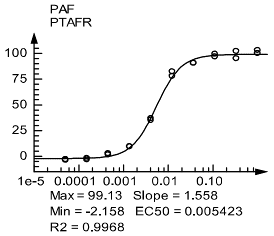
Prostaglandin E2	Arrestin	Agonist	PTGER2	EC50	0.42614	1.04	0.5	106.1	108.69	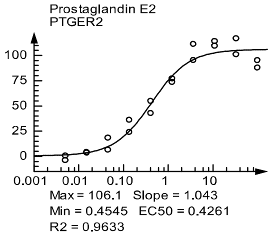

Compound Name	Assay Name	Assay Format	Assay Target	Result Type	RC50 (uM)	Hill	Curve Bottom	Curve Top	Max Response	Result Graph
Prostaglandin E2	Arrestin	Agonist	PTGER3	EC50	0.003581	1.25	-0.5	98.9	101.09	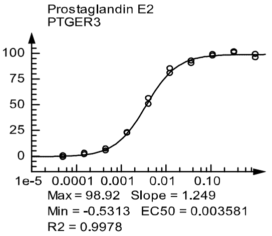
Prostaglandin E2	Arrestin	Agonist	PTGER4	EC50	0.00076524	1.36	0	97.6	100.95	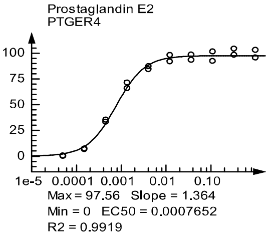
Cloprostenol	Arrestin	Agonist	PTGFR	EC50	0.012248	0.99	0	95.9	101.95	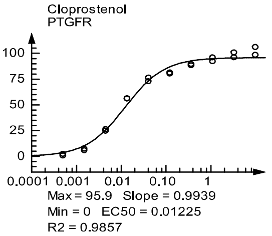
Beraprost	Arrestin	Agonist	PTGIR	EC50	0.2894	0.95	0	99.6	102.94	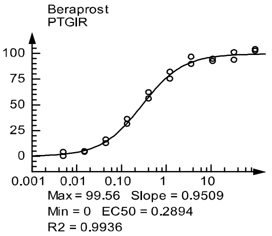
PTH(1-34)	Arrestin	Agonist	PTHR1	EC50	0.0010943	2.08	0	98.1	101.02	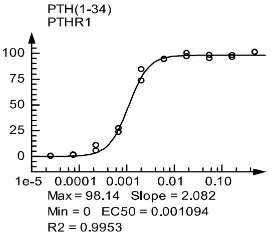
TIP-39	Arrestin	Agonist	PTHR2	EC50	0.0011596	1.97	0	97.9	98.773	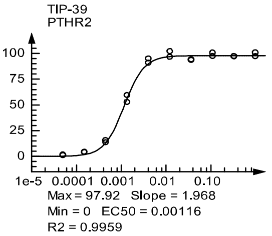
Relaxin-3	Arrestin	Agonist	RXFP3	EC50	0.005712	1.01	-6.5	102	101.9	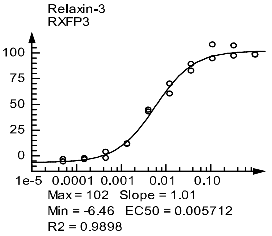

Compound Name	Assay Name	Assay Format	Assay Target	Result Type	RC50 (uM)	Hill	Curve Bottom	Curve Top	Max Response	Result Graph
Secretin	Arrestin	Agonist	SCTR	EC50	0.00065974	1.99	-0.1	100	93.727	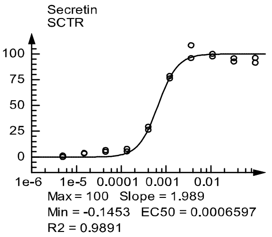
Somatostatin 28	Arrestin	Agonist	SSTR1	EC50	0.0059721	0.65	-3	95.5	92.857	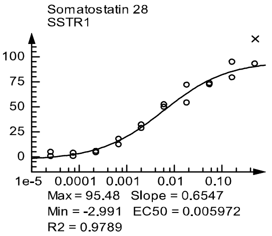
Somatostatin 28	Arrestin	Agonist	SSTR2	EC50	0.0029291	1.57	2.3	100	111.01	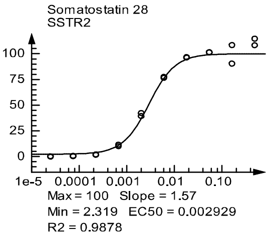
Tyr-SST 14	Arrestin	Agonist	SSTR3	EC50	0.015542	0.95	-2.2	100	100	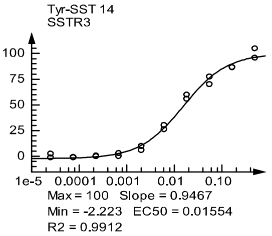
Somatostatin 28	Arrestin	Agonist	SSTR5	EC50	0.022836	1.25	-1.6	102.8	104.21	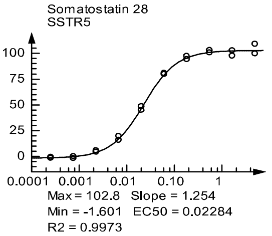
Substance P	Arrestin	Agonist	TACR1	EC50	0.0048015	2.48	1.7	100	102.59	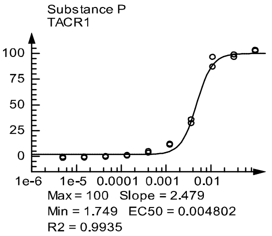
Substance P	Arrestin	Agonist	TACR2	EC50	0.071399	1.04	4.6	95	106.57	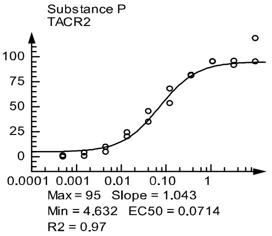

Compound Name	Assay Name	Assay Format	Assay Target	Result Type	RC50 (uM)	Hill	Curve Bottom	Curve Top	Max Response	Result Graph
Substance P	Arrestin	Agonist	TACR3	EC50	0.017672	1.19	0	98.4	100.39	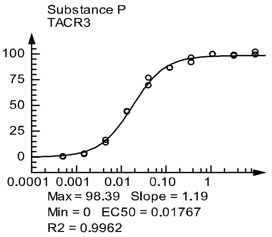
I-BOP	Arrestin	Agonist	TBXA2R	EC50	0.013512	0.86	0	100.6	100.31	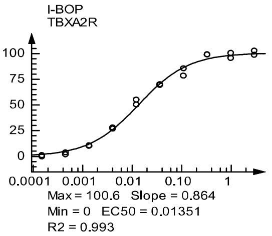
TRH	Arrestin	Agonist	TRHR	EC50	0.0016363	1.09	1.1	100	100.54	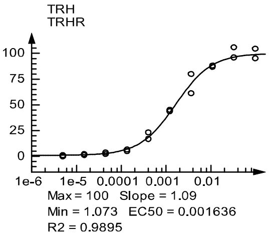
TSH	Arrestin	Agonist	TSHR(L)	EC50	0.050591	1.28	4.5	100	103.8	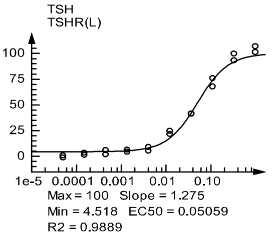
Urotensin II	Arrestin	Agonist	UTR2	EC50	0.0011246	1.15	0	96.2	101.17	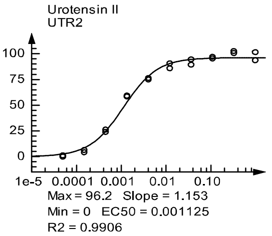
VIP	Arrestin	Agonist	VIPR1	EC50	0.0018607	1.85	0	98.9	98.836	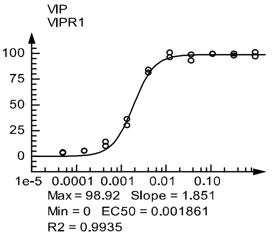
VIP	Arrestin	Agonist	VIPR2	EC50	0.0026929	2.36	-0.5	100	101.19	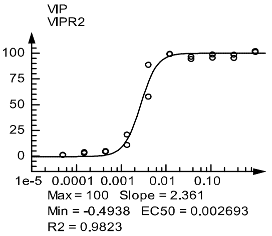
